# Microphysical Modeling of Carbonate Fault Friction at Slip Rates Spanning the Full Seismic Cycle

**DOI:** 10.1029/2020JB021024

**Published:** 2021-03-25

**Authors:** Jianye Chen, A. R. Niemeijer, Christopher J. Spiers

**Affiliations:** ^1^ State Key Laboratory of Earthquake Dynamics Institute of Geology China Earthquake Administration Beijing China; ^2^ HPT Laboratory Department of Earth Sciences Utrecht University Utrecht The Netherlands; ^3^ Now at Geoscience & Engineering Department Delft University of Technology Delft The Netherlands

**Keywords:** dynamic fault weakening, earthquake/rupture modeling, frictional heating, high‐velocity friction, seismic cycle, superplastic flow

## Abstract

Laboratory studies suggest that seismogenic rupture on faults in carbonate terrains can be explained by a transition from high friction, at low sliding velocities (*V*), to low friction due to rapid dynamic weakening as seismic slip velocities are approached. However, consensus on the controlling physical processes is lacking. We previously proposed a microphysically based model (the “Chen–Niemeijer–Spiers” [CNS] model) that accounts for the (rate‐and‐state) frictional behavior of carbonate fault gouges seen at low velocities characteristic of rupture nucleation. In the present study, we extend the CNS model to high velocities (1 mm/s ≤ *V* ≤ 10 m/s) by introducing multiple grain‐scale deformation mechanisms activated by frictional heating. As velocity and hence temperature increase, the model predicts a continuous transition in dominant deformation mechanisms, from frictional granular flow with partial accommodation by plasticity at low velocities and temperatures, to grain boundary sliding with increasing accommodation by solid‐state diffusion at high velocities and temperatures. Assuming that slip occurs in a localized shear band, within which grain size decreases with increasing velocity, the model results capture the main mechanical trends seen in high‐velocity friction experiments on room‐dry calcite‐rich rocks, including steady‐state and transient aspects, with reasonable quantitative agreement and without the need to invoke thermal decomposition or fluid pressurization effects. The extended CNS model covers the full spectrum of slip velocities from earthquake nucleation to seismic slip rates. Since it is based on realistic fault structure, measurable microstructural state variables, and established deformation mechanisms, it may offer an improved basis for extrapolating lab‐derived friction data to natural fault conditions.

## Key Points

- We extend a microphysical friction model from earthquake nucleation to seismic slip velocities
- The extended model predicts steady‐state and transient friction
- Model results reproduce high‐velocity friction experiments on simulated calcite fault gouges

## 1 Introduction

Laboratory fault friction experiments on a wide range of rock materials show a broadly similar dependence of friction coefficient on sliding velocity (*V*) over the full range of sliding velocities expected in transitioning from earthquake rupture nucleation (∼1 μm/s) to propagation (∼1 m/s). Slow sliding (μm/s) produces high friction and rate‐and‐state‐dependent behavior, with steady‐state friction coefficient increasing with *V* until a peak at around 1–10 cm/s, beyond which rapid weakening occurs as seismic velocities are attained (∼1 m/s, Buijze et al., 2017; Di Toro et al., 2011; Ferri et al., 2011; Kohli et al., 2011; Reches & Lockner, 2010). Various dynamic weakening mechanisms have been proposed to explain this sharp drop in friction. These include melt lubrication, flash heating, silica gelification, nanopowder lubrication, thermal or thermochemical pressurization, and pore water vaporization (J. Chen, Niemeijer, Yao, et al., 2017; De Paola et al., 2011; Di Toro et al., 2011; Han et al., 2007; A. Niemeijer et al., 2012; Reches & Lockner, 2010; Rice, 2006).

Following the highly destructive moderate to large earthquakes that have occurred in carbonate‐dominated terrains in the last 10–15 years (e.g., the 2008 Mw 7.9 Wenchuan earthquake, China; the 2009 Mw 6.1 L'Aquila earthquake; and the 2016 Mw 6.5 Norcia Earthquake, Italy), the frictional behavior of carbonate(‐bearing) faults has attracted much attention, with a view to providing appropriate data for modeling earthquake nucleation and seismic rupture propagation in such terrains. Experiments have been performed at a wide range of velocities and stress‐temperature conditions and have revealed a roughly similar dependence of frictional strength on sliding velocity to that seen in silicate rocks. However, in contrast to silicate‐dominated rocks, which often show evidence for melt‐related dynamic weakening at high velocities, melting is not observed. Decomposition of carbonate minerals (producing CO_2_ and oxides) has been widely reported in both laboratory and natural carbonate fault zones (De Paola et al., 2011; Han et al., 2007; Violay et al., 2013) but cannot account for the fact that dynamic weakening in experiments occurs at short displacements (<<1 m) before temperatures reach high enough values to allow substantial decarbonation (De Paola et al., 2015; Spagnuolo et al., 2015). Instead, microstructural analysis has revealed that dynamic weakening is more likely caused by the formation of a dense, nanogranular, viscous, or “superplastic” shear band of a few micrometers to tens of micrometers in thickness (e.g., Boneh & Reches, 2018; Demurtas, Smith, Prior, Spagnuolo, et al., 2019; De Paola et al., 2015; Green et al., 2015; Pozzi et al., 2018). While this may explain the dynamic weakening seen in laboratory experiments on “lab‐dry” carbonate faults at high velocities, that is, above ∼5 cm/s, a sharp peak in strength seen at slightly lower velocities (1 mm/s < *v* < 5 cm/s, e.g., Smith et al., 2015) has not been accounted for. Moreover, previous studies have not addressed how (super)plastic, viscous‐flow processes supersede the mechanisms operating at lower velocities, where shearing of fault gouge is dominated by frictional granular flow accompanied by diffusive mass transport via adsorbed moisture films or by limited crystal plastic flow (Verberne et al., 2013; Verberne, Plümper, et al., 2014). In addition, while empirical rate‐and‐state‐dependent friction (RSF) and microphysically based models (e.g., Dieterich, 1979; A. R. Niemeijer & Spiers, 2007) are available for describing the low‐velocity (LV) frictional behavior of faults in carbonates (Carpenter et al., 2014; J. Chen & Spiers, 2016; Scuderi et al., 2013; Verberne et al., 2015; Weeks & Tullis, 1985), mechanistically based models addressing the full range of slip rates encountered in progressing from rupture nucleation to seismic slip are limited to recent steps in this direction by Aharonov and Scholz (2018). A low‐to‐high‐velocity description of this type is essential for numerically modeling the rupture nucleation and propagation process (e.g., Beeler, 2009; Weeks, 1983) but up to now has been limited to the combination of RSF descriptions at low slip rates with thermal pressurization or flash heating/melting in the dynamic weakening range (Aharonov & Scholz, 2018; Noda & Lapusta, 2010; Noda et al., 2009; Spagnuolo et al., 2016), with no independent evidence that these mechanisms are valid, at least for carbonate faults.

In this study, we extend our previous microphysical model, developed for frictional shearing of fault gouges at low velocities (<1 mm/s) where dilatant granular flow plus water‐assisted diffusive mass transfer have been inferred to control the deformation behavior of wet granular calcite (A. R. Niemeijer & Spiers, 2007; J. Chen & Spiers, 2016, hereafter referred to as the CNS model), to seismic velocities (∼1 m/s) where shear heating causes a continuous transition to (super)plastic viscous flow—as proposed by De Paola et al. (2015). We show that the full spectrum of frictional behavior seen in low‐ to high‐velocity friction (HVF) experiments conducted on carbonate gouges at room humidity and temperature (RHT) can be reproduced in a single model framework simply by allowing for the effects of frictional heating on the rates of well‐known, thermally activated processes operating at the grain scale. We then proceed to discuss the implications of our results for earthquake rupture in carbonate terrains.

## 2 Background on HVF in Carbonates at Room Conditions

**Figure 1:**
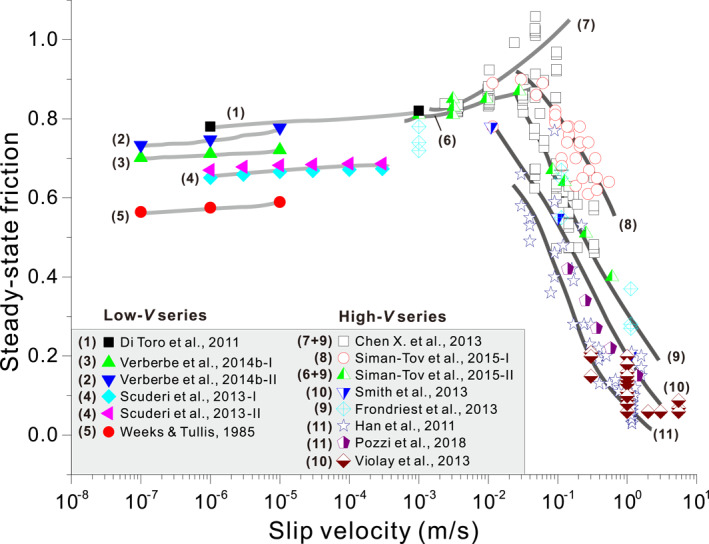
Compilation of steady‐state friction coefficients of simulated gouges or bare surfaces of carbonate rocks sheared at slip rates from 0.1 μm/s to 1.4 m/s under room humidity and temperature conditions. The HV series of experiments were performed using rotary‐shear apparatuses under relatively low normal stresses (*σ*_*n*_ *= *1–26 MPa), while the LV series were conducted at high normal stress (*σ*_*n*_ *= *50–100 MPa) using conventional shear apparatuses. Significant increases in temperature (>100°C) are expected for experiments sheared at a nominal slip rate greater than 0.01 m/s and *σ*_*n*_ ≥ 10 MPa (e.g., Demurtas, Smith, Prior, Brenker, et al., 2019). *LV series*—Di Toro et al. (2011): on bare surfaces at *σ*_*n*_ = 1.25 MPa; Weeks and Tullis (1985): on bare surfaces of dolomite at *σ*_*n*_ = 75 MPa, with ∼40‐μm‐thick gouge developed; Verberne, Spiers, et al. (2014)‐I: on limestone fault gouges at *σ*_*n*_ = 50 MPa; Verberne, Spiers, et al. (2014)‐II: on calcite fault gouges at *σ*_*n*_ = 50 MPa; Scuderi et al. (2013)‐I: on dolomite gouges at *σ*_*n*_ = 75 MPa; and Scuderi et al. (2013)‐II: on dolomite gouges at *σ*_*n*_ = 100 MPa. *HV series*—Siman‐Tov et al. (2015)‐I: on bare surfaces of the KG samples sheared at *σ*_*n*_ = 1–2 MPa; Siman‐Tov et al. (2015)‐II: on bare surfaces of the DG samples sheared at *σ*_*n*_ = 1–2 MPa; X. Chen et al. (2013): on dolomite gouges at *σ*_*n*_ up to 7 MPa; Smith et al. (2013): on carbonate gouges at *σ*_*n*_ = 17.3–21.5 MPa; Frondriest et al. (2013): on carbonate gouges at *σ*_*n*_ = 17.3 MPa; Han et al. (2011): mostly on bare marble surfaces at *σ*_*n*_ = 2–15 MPa; Pozzi et al. (2018): on calcite gouges at *σ*_*n*_ = 25 MPa; and Violay et al. (2013): on Carrara marble surfaces at *σ*_*n*_ = 10–20 MPa. The thick lines show the interpreted trends of steady‐state friction with velocity in different ranges. HV, high velocity; LV, low velocity.

To extend the CNS model from low to high fault sliding velocities, our first step is to review available data on the frictional behavior of simulated carbonate fault gouges across a wide velocity spectrum. Figure 1 shows a compilation of steady‐state friction coefficients obtained for simulated carbonate fault gouges (and initially bare rock surfaces) in experiments performed at RHT and at velocities (*V*) ranging from 0.1 μm/s to 6.5 m/s. Data obtained at low velocities (LV, namely, 0.1 μm/s < *V* < 1 mm/s) are derived from direct shear experiments at relatively high normal stress (*σ*_*n*_ = 50–100 MPa), while the high‐velocity (HV) data (namely, *V* ≥ 1 mm/s) were obtained using HV rotary‐shear methods, usually at relatively low normal stress (1–26 MPa). At LV, the steady‐state friction coefficient falls between 0.56 and 0.8 at RHT, with all data consistently displaying *V*‐strengthening behavior with a rate‐dependent RSF parameter, (*a*–*b*), of 0.003–0.007 (thick gray Lines 1–5, Figure 1). The HVF data show similar scatter between studies and can be divided into two regimes. At 1 mm/s to 0.1 m/s, a sharp peak in frictional strength occurs (Lines 6–7). At *V* ≥ 0.1 m/s, a sharp dynamic weakening occurs at consistent weakening rates as *V* increases (i.e., similar slopes of dark gray Lines 8–11). Different data sets in this regime tend to show that the frictional strength is lower at higher normal stress (see the Figure S1, where the same data sets are plotted as a function of power density, Boneh et al., 2013). To define the key ingredients for the extended CNS model, we shall now identify the microphysical deformation processes operating in these different velocity ranges.

### 2.1 Mechanisms Controlling Friction at Low Velocities (ca., *V* ≤ 1 mm/s)

In LV experimental studies on dry carbonate rocks, shear deformation tends to localize to a thin and highly comminuted slip zone showing a cataclastic microstructure with submicrometer matrix grain size (e.g., Scuderi et al., 2013; also seen in the short‐stage microstructure in a HVF experiment,; Smith et al., 2015). Cataclastic flow, giving way to granular flow (frictional grain neighbor swapping) beyond the comminution limit of 0.1–1.0 µm (cf., Sammis & Ben‐Zion, 2008), was inferred to be key shearing mechanisms at these velocities under both wet and dry conditions (Smith et al., 2015; Verberne, Spiers, et al., 2014). Since calcite is relatively ductile at low temperatures, crystal plastic creep mechanisms such as mechanical twinning and dislocation glide are likely to be active at grain contacts, even at room temperature (De Bresser & Spiers, 1997). Moreover, the presence of the absorbed water cannot be ruled out, even in nominally dry experiments, so that water‐film‐assisted deformation processes, such as pressure solution creep, should be active too, especially where the grain size is small (Verberne, Spiers, et al., 2014), although the kinetics might be different from those in the fully wet experiments. On this basis, we propose that calcite friction at low velocities and at RHT is controlled by granular flow plus enhanced “plastic” creep at grain contacts by dislocation or water‐assisted diffusive processes. In a recent study, J. Chen et al. (2020) incorporated granular flow plus a general grain‐scale creep law into the CNS model to predict the steady‐state frictional behavior of dry calcite gouge at low temperatures and slip rates from 0.1 μm/s to 1 mm/s. The *V*‐strengthening friction results obtained are similar to the experimental data obtained at low velocities (e.g., Figure 1), supporting the mechanisms that we infer here.

### 2.2 Mechanisms Causing Marked *V*‐Strengthening at 1 mm/s ≤ *V* ≤ 0.1 m/s

**Figure 2:**
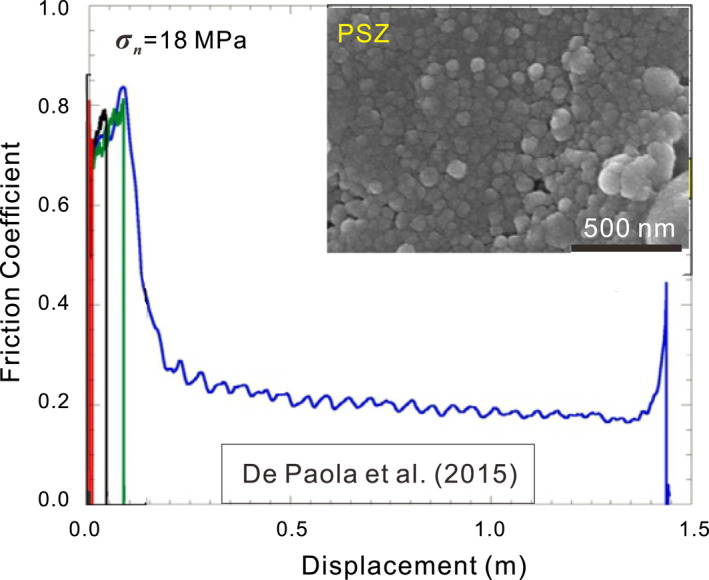
Typical results from an HVF experiment on calcite gouge sheared at 1.0 m/s and 18 MPa normal stress with 1.45 m shear displacement (De Paola et al., 2015). The inset presents the scanning electron microscopy image of the principal slip zone (PSZ), consisting of compact aggregates of nanocrystalline calcite with a polygonal texture (see Figure 8 of De Paola et al., 2015). HVF, high‐velocity friction.

In contrast to the behavior seen at lower velocities, steady‐state friction data obtained at velocity range 1 mm/s to 0.1 m/s demonstrate an exceptionally high rate of *V*‐strengthening (Figure 1). This correlates with a marked transient peak strength developed in friction versus displacement curves during the acceleration stage of experiments performed in this velocity range (cf., Figure 2). We expect that the deformation mechanisms operating in this *V*‐range are similar to those at low velocities, that is, granular flow involving frictional grain neighbor swapping, with partial accommodation by a creep mechanism operating at grain contacts (Demurtas, Smith, Prior, Brenker, et al., 2019; Demurtas, Smith, Prior, Spagnuolo, et al., 2019). However, the onset of shear heating in this *V*‐range (up to 0.1 m/s), even at the modest normal stresses applied in HVF experiments (ca., 5 MPa), must provide an increased contribution to grain contact or wider grain‐scale deformation by thermally activated dislocation or diffusion creep processes (Demurtas, Smith, Prior, Spagnuolo, et al., 2019; Smith et al., 2013, 2015; Spagnuolo et al., 2015). Since shear deformation remains frictional, this must be dominated by cataclastic/granular flow rather than any creep process. However, as the dilatancy angle characterizing granular shear flow is generally low (Budhu, 2010), associated gouge dilatation rates will also be low, so that even modest thermal activation of creep at the grain scale will tend to produce compaction to lower porosity levels, thus increasing frictional strength. On this basis, and taking into account the widely reported observation of continued compaction and moderate temperature rise in friction experiments performed in the range 1 mm/s ≤ *V* ≤ 0.1 m/s (Demurtas, Smith, Prior, Brenker, et al., 2019; Pozzi et al., 2018; Rempe et al., 2017), we propose that the marked *V*‐strengthening observed in this range is due to compaction creep of the principal slip zone (PSZ) enhanced by temperature rise due to frictional heating.

### 2.3 Mechanisms Controlling Dynamic Frictional Weakening (*V* ≥ 0.1 m/s)

At *V* ≥ 0.1 m/s, experiments on lab‐dry carbonate fault gouges show a remarkable decrease in steady‐state friction (Figure 1). Typical experiments at these velocities show a transient peak strength, at short displacements, followed by dynamic weakening, with increasing displacement, toward nominal steady‐state friction coefficients as low as 0.2 (Figure 2). As discussed in Section 1, weakening mechanisms such as melting, extensive decarbonation, and thermal or thermochemical pressurization can be excluded. Flash heating offers a possible alternative mechanism for fast incipient weakening (Aharonov & Scholz, 2018; De Paola et al., 2011; Rice, 2006; Tisato et al., 2012). In this case, frictional heat is generated, by high‐*V* sliding at a few highly stressed contact points, faster than it can diffuse away so that asperity temperatures become high enough to degrade their strength and reduce friction (Spagnuolo et al., 2015). This mechanism might be important for bare rock friction but it is probably minor in gouge‐filled slip zones, due to the ultra‐fine grain sizes (<100 nm) and lower intergranular sliding velocities involved (due to multiple grain contacts within the PSZ, De Paola et al., 2015). At the same time, though, some role of flash heating or decarbonation on the very early stage of weakening, before or during nanogouge formation, cannot be fully excluded. Following Han et al. (2011), pronounced weakening could otherwise be caused by rolling of nanoparticles, a form of powder lubrication, inferred to control friction at near‐seismic velocities and above (X. Chen et al., 2013; Reches & Lockner, 2010). However, Yao et al. (2016) demonstrated that powder lubrication alone cannot account for low dynamic friction values in rocks without invoking a high temperature and suggested that thermally activated processes at grain boundaries in nanogranular wear products may dominate mechanical behavior.

As proposed by De Paola et al. (2015) and Green et al. (2015), the most likely mechanism operating at high strain rates ($\dot{\gamma }\;$>10^3^ s^−1^) and temperatures in the fine‐grained PSZ of a dry carbonate‐hosted fault is “grain‐size‐sensitive flow” involving solid‐state “grain boundary sliding (GBS) with accommodation by diffusion,” provided decarbonation is minor (i.e., with short displacement). In materials science, this mechanism of GBS with diffusional accommodation is often referred to as “superplastic flow” because its viscous character allows large ductile strains to be achieved in unconfined extension tests, especially in metals (where accommodation can also involve dislocation motion and grain boundary migration [Gifkins, 1976; Langdon, 1994]).

Microstructural analyses of carbonates sheared in HV friction experiments have revealed a shiny layer of a few tens of micrometers in thickness, usually surrounded by well‐sintered zones at the sides (Pozzi et al., 2018). This layer is inferred to be the PSZ and consists of a continuous layer of nanocrystalline calcite with a nominal grain size of 10–600 nm in diameter, showing polygonal grain boundaries and high‐angle junctions (inset of Figure 2, De Paola et al., 2015). Field studies of natural carbonate‐hosted faults demonstrate the existence of well‐polished, glossy “surfaces,” usually termed “fault mirrors,” that consists of tightly packed nanoparticles with grain and grain boundary structures similar to those seen in the experiments (Fondriest et al., 2013; Ohl et al., 2020; Siman‐Tov et al., 2013; Smith et al., 2013). These microstructural characteristics of both the experimental and natural PSZs are indeed similar to those observed in high‐pressure and ‐temperature (HPT) experiments on fine‐grained carbonate rocks for which superplastic behavior has been inferred (Herwegh et al., 2003; Rutter et al., 1994; Schmid et al., 1977; Walker et al., 1990) and are consistent with expectations based on microphysical models for superplasticity (e.g., Ashby & Verrall, 1973).

### 2.4 De Paola et al.'s Model for Dynamic Frictional Weakening

Models for high‐temperature deformation of a crystalline solid by GBS accommodated by diffusion or by dislocation motion generally result in a “viscous” flow law of the form (Ashby & Verrall, 1973):

(1a) $${\dot{\gamma }}_{pl}=A\left(\frac{D_{o}Gb}{kT}\right){\left(\frac{\tau }{G}\right)}^{n}{\left(\frac{b}{d}\right)}^{m}\;\text{exp}\left(-\frac{E_{a}}{RT}\right).$$

Here, *A* is a dimensionless geometrical constant,τ is the differential or shear stress, *G* is the shear modulus, *b* is a Burgers vector, *D*_*o*_ is the thermal coefficient, *E*_*a*_ the activation energy for the relevant diffusion process (volume or grain boundary), *T* is absolute temperature, and *k* is Boltzmann's constant. GBS is considered to involve the motion of grain boundary dislocations or viscous sliding on thin melt or amorphous layers present in grain boundary (GB)'s (Ashby & Verrall, 1973; Gifkins, 1976; Hynes & Doremus, 1996) and is therefore nonfrictional. Possible accommodation processes include solid‐state grain boundary diffusion (*n* = 1 and *m* ≈ 3, Ashby & Verrall, 1973), dislocation slip (*n* = 2 and *m* = 2, Gifkins, 1976), and recrystallization, as well as diffusion through any GB melt or glassy film (e.g., Hynes & Doremus, 1996). Equation 1a is often simplified into the general form for empirically describing creep of dense crystalline solids (e.g., Herwegh et al., 2003; Schmid et al., 1977), written

(1b) $${\dot{\gamma }}_{pl}=A_{pl}\;\text{exp}\left(-\frac{E_{pl}}{RT}\right)\frac{\tau^{n}}{d^{m}}.$$

The main difference between this and 1a lies in the composite preexponential factor *A*_*pl*_ and the temperature‐compensated value of the apparent activation energy *E*_*pl*_.

De Paola et al. (2015) calculated the steady‐state flow stress of a simulated calcite gouge during HV sliding, taking realistic grain sizes (*d*) and temperatures (*T*) obtained from experiments, using Equation 1b as established by Schmid et al. (1977) for fine‐grained calcite aggregates at HPT conditions (*n* = 1.7, *m* = 3). The results gave flow stresses of a few MPa, which are consistent with the shear stress levels measured in the HVF experiments reported by De Paola et al. (see also experiments by Demurtas, Smith, Prior, Spagnuolo, et al., 2019).

To describe fault sliding resistance over the full range of slip rates spanning earthquake nucleation to seismic slip velocities shown in Figure 1, a model framework is needed that links the De Paola “viscous‐flow” model for a dense, nanogranular, carbonate fault rock to the LV regime where gouges possess a porous, cataclastic microstructure and deform by brittle grain‐size reduction, granular flow (e.g., Scuderi et al., 2013; Smith et al., 2015). Moreover, to simulate the full evolution of a seismic slip event (including slip acceleration and deceleration), the sought model must capture both steady‐state and transient behavior, as seen in HVF experiments (Figure 2).

## 3 The CNS Model: Its Range of Applicability to Date

In this section, we summarize our existing microphysical model (the CNS model), originally developed for friction of a shearing granular fault gouge at low (subseismic) velocities where (nano)granular cataclastic/frictional flow with partial accommodation by intergranular pressure solution (IPS) control deformation (J. Chen & Spiers, 2016; A. R. Niemeijer & Spiers, 2007), showing how it breaks down at seismic velocities. At these high velocities, shear heating becomes significant so that other mechanisms, such as nonfrictional GBS with accommodation by crystal plasticity and/or solid‐state diffusional flow, take over. A detailed derivation of the CNS model is presented by J. Chen and Spiers (2016). Here, we summarize only the key elements presently needed.

### 3.1 Elements of the CNS Model for LV Friction

**Figure 3:**
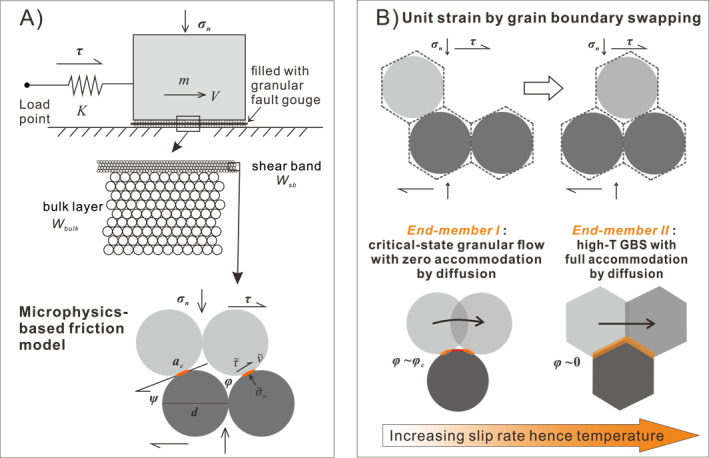
(a) The spring‐slider fault model representing a laboratory fault filled with simulated carbonate gouges which show slip localization, and the representative unit cell characterizing the microstructure of the grain pack under active shear deformation. (b) Schematic model illustrating deformation due to a unit grain neighbor swapping event for two end‐member cases. A transition occurs with increasing slip rate and hence temperature from *End‐member I* (critical‐state granular flow with frictional sliding between grains and zero accommodation by diffusion) to *End‐member II* (high‐temperature GBS with full accommodation by diffusion hence zero porosity). GBS, grain boundary sliding.

The CNS model assumes a fault filled with a granular gouge as represented in the 2‐D section shown in Figure 3a. As widely observed in both natural and laboratory fault zones in carbonates, deformation is assumed to localize into a thin, finer‐grained shear band developed within or at the boundary of the gouge layer. The shear band and bulk sample are modeled assuming them to be composed of grains represented by cylinders or spheres with the same general packing but with different mean diameters representing the finer shear band and coarser bulk grain sizes (Figure 3a, middle panel).

The microstructure of the shear band is described using a 4‐grain unit cell (Figure 3a, lower panel). Porosity (*φ*), dilatancy angle (*ψ*), and contact area (*a*_*c*_) within the shear band form a set of three, geometrically interrelated state variables, characterizing the internal microstructural state. These are given by the approximate relations (A. R. Niemeijer & Spiers, 2007):

(2a) $$\tan\;\psi =2H\left(\varphi_{c}-\varphi \right)$$

and

(2b) $$a_{c}=2\pi d^{2}\left(\varphi_{c}-\varphi \right)/z,$$

where *H* is a constant related to the packing geometry of the grains, $\varphi_{c}$ is the critical‐state porosity for granular flow, *d* is nominal grain diameter, and *z* is the average coordination number. The microstructural state and overall frictional strength of the gouge are controlled by the porosity within the shear band and hence that dilatation angle. These are in turn controlled by competition between dilatation due to granular flow and compaction by time‐dependent, thermally activated creep at grain contacts (assumed to be driven by effective normal stress only).

As derived from kinematic and thermodynamic considerations (J. Chen & Spiers, 2016), the equations governing the microstructural state and shear strength of the model shear band (Figure 3a) are

(3a) $$\frac{\dot{\varphi }}{\left(1-\varphi \right)}={\dot{\gamma }}_{gr}\;\tan\;\psi -{\dot{\varepsilon }}_{pl}$$

(3b) $$\tau =\sigma_{n}\frac{\tilde{\mu }+\tan\;\psi }{1-\tilde{\mu }\;\tan\;\psi }$$

In these equations, $\dot{\gamma }$ and $\dot{\varepsilon }$ indicate the shear and normal strain rates, respectively. Subscript *pl* indicates strain rates due to any “plastic” creep mechanism, while *gr* indicates contributions by granular flow. Equation 3a is a “state evolution equation,” expressing porosity change and hence deformation in the fault‐normal direction (i.e., volumetric strains) in terms of the dilatation strain rate ${\dot{\varepsilon }}_{gr}={\dot{\gamma }}_{gr}\;\tan\;\psi$ due to granular flow and the compaction strain rate $\;{\dot{\varepsilon }}_{pl}\;$ by an arbitrary “plastic” creep mechanism. Equation 3b is a “friction law” giving the shear strength of a sliding gouge zone in terms of the grain boundary friction $\;\tilde{\mu }\;$ and the contribution to shear strength due to the intergranular dilatation angle (ψ).

GB friction is assumed to be cohesion free and hence given by $\tilde{\mu }=\tilde{\tau }/{\tilde{\sigma }}_{n}$, where $\tilde{\tau }$ and ${\tilde{\sigma }}_{n}$ are shear and normal stresses acting on sliding grain contacts, respectively (Figure 3a). Based on lattice‐scale frictional interactions (J. Chen & Spiers, 2016), $\tilde{\mu }$ is expected to be intrinsically rate strengthening and can be reformulated in terms of the strain rate due to granular flow as follows:

(4) $$\tilde{\mu }={\tilde{\mu }}^{*}+a_{\tilde{\mu }}\;\ln\left({\dot{\gamma }}_{gr}/{\dot{\gamma }}_{gr}^{*}\right).$$

The sensitivity parameter $a_{\tilde{\mu }}$ is given as $a_{\tilde{\mu }}=kT/\sigma_{l}\Omega$, where *T* is temperature, *k* the Boltzmann constant, Ω the activation volume, and $\sigma_{l}$ the local stress supported by individual asperity contacts.

Granular flow is assumed to occur preferentially in the localized shear band such that shear deformation parallel to the fault plane results from the parallel operation of granular flow, at the rate $V_{gr}=W_{sb}{\dot{\gamma }}_{gr}$, and plastic creep process in both the shear band and bulk layer, at the rate $V_{pl}=W_{sb}{\dot{\gamma }}_{pl}^{sb}+W_{bulk}{\dot{\gamma }}_{pl}^{bulk}$. Expressed in terms of fault slip velocity *V*, this can be written as

(5) $$V=W_{sb}{\dot{\gamma }}_{gr}+W_{sb}{\dot{\gamma }}_{pl}^{sb}+W_{bulk}{\dot{\gamma }}_{pl}^{bulk}.$$

Here, the subscript or superscript *sb* and *bulk* represent the shear band and bulk layer, respectively.

### 3.2 Predictions of the Original CNS Model and Its Breakdown at High Velocities

**Figure 4:**
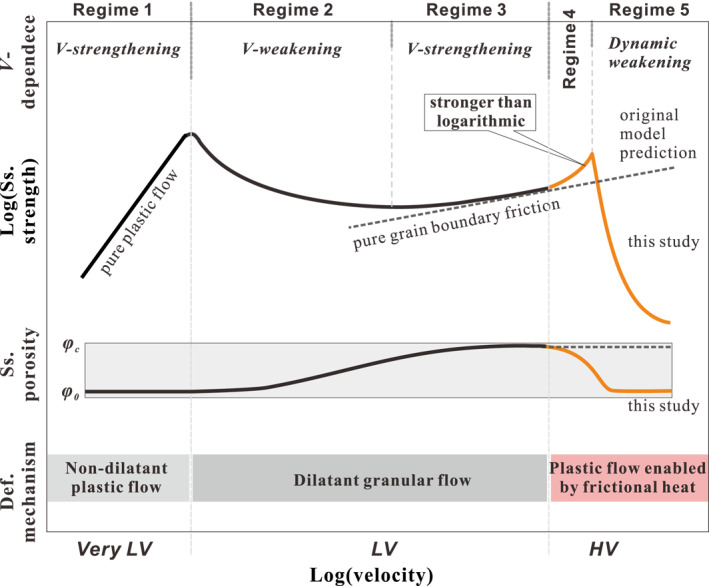
Schematic diagram showing the general picture of steady‐state (ss.) frictional strength and porosity as a function of logarithmic velocity for a fault filled with granular material, as embodied in the CNS model (modified after J. Chen, Niemeijer, & Spiers, 2017; J. Chen & Niemeijer , 2017). Deformation is generally divided into five regimes (*Regimes 1–5*), characterized by the varied *V*‐dependences of friction. See a detailed description of the deformation regimes and the corresponding controlling deformation (def.) mechanisms in the text. CNS, Chen–Niemeijer–Spiers.

Figure 4 shows a total of five steady‐state deformation regimes (*Regimes 1–5*), occurring across three ranges of slip rate, namely the “very‐*LV*” range (containing Regime 1) the “*LV*” range (containing Regimes 2 and 3) and the “*HV*” range (containing Regimes 4 and 5). The first three regimes are predicted by the original CNS model (J. Chen, Niemeijer, & Spiers, 2017; J. Chen & Niemeijer, et al., 2017). At very‐low velocities (*Regime 1*), fault‐parallel plastic flow is fast enough to fully achieve the imposed shear deformation without intergranular dilatation ($V_{gr}\ll V_{pl}$), causing markedly *V*‐strengthening behavior, that is, fully plastic flow (cf., Figure 3b). As *V* increases, plastic flow is too slow to accommodate the imposed shear rate, so that grain boundaries are forced to slide and dilate, with the result that granular flow starts to dominate over plastic flow ($V_{gr}\gg V_{pl}$). At these shear rates, the dilatancy associated with granular flow increases porosity until a steady state is reached where dilatation is balanced by compaction due to creep occurring at grain contacts. This increase in porosity with increasing velocity leads to a decrease in dilatation angle ψ at steady state (refer to Figure 3) and hence to a reduction in steady‐state shear strength via Equation 3b, causing a transition to *V*‐weakening granular flow with a sensitivity that decreases with increasing *V* (*Regime 2*). At the same time, granular flow involves grain boundary slip, which is intrinsically rate strengthening (Equation 4). Hence, as *V* increases further and granular flow and intergranular slip becomes progressively faster, the rate strengthening nature of grain boundary friction causes a further transition from *V*‐weakening to *V*‐strengthening (*Regime 3*).

In the “very‐*LV*” and “*LV*” ranges, analytical solutions for shear strength and porosity have been obtained for *Regime 1* and *Regimes 2* and *3* by J. Chen, Niemeijer, & Spiers (2017) and J. Chen & Niemeijer (2017). The numerical solution presented by J. Chen, Niemeijer, & Spiers (2017) and J. Chen & Niemeijer (2017) for strength in the transition from *Regime 1* to *2*, which is essentially the ductile‐to‐brittle transition (Kawamoto & Shimamoto, 1992), represents parallel operation of plastic flow and granular flow. It thus yields a peak in shear strength that falls below the analytical solutions for these mechanisms at the transitional velocity (cf., Reinen et al., 1992). This portion of the model has recently been successfully applied to explain the strength profile of a calcite gouge sheared at 550°C and across slip rates spanning almost 6 orders of magnitude (J. Chen et al., 2020).

As *V* increases to even higher values, and if no new mechanisms are activated, deformation will approach being fully by steady‐state granular flow, or “critical‐state granular flow” (Paterson, 1995), occurring at an asymptotic maximum or critical‐state porosity (*φ* ≈ *φ*_*c*_), whereby the dilatation angle is zero and shear strength is controlled only by velocity‐strengthening grain boundary friction (see the gray dashed line, Figure 4). However, laboratory observations at high velocities (Figure 1) show that this prediction by the original CNS model ultimately breaks down at *V* > 1 mm/s, giving way to a rapid increase in friction defining the onset of *Regime 4* in Figure 4, followed by dynamic weakening in *Regime 5* (see Figure 1). We propose that this is due to the activation of another, thermally activated deformation mechanism, first causing enhanced compaction creep (*Regime 4*) and then enhanced shear creep (*Regime 5*), similar to that proposed by De Paola et al. (2015). Our expectation is that as temperature increases and grain size decreases in the HV shearing field (see Section 2), shear deformation within the PSZ will experience a transition in mechanism, from critical‐state (dilatant) granular flow involving frictional GBS, as described by the original CNS model in *Regime 3* (Figure 4), to dilatant granular flow (high‐temperature GBS/cavitation) operating in competition with viscous compaction (*Regime 4*) and shear (*Regime 5*)—by high temperature, solid‐state diffusion (Figure 3b). In the following, we extend the CNS model to take these high‐temperature processes into account, along with the effects of grain‐size reduction observed in HVF experiments (De Paola et al., 2015).

It is important to note that the schematic profiles predicted in Figure 4 illustrate the evolution of shear strength and porosity in a dry gouge material, such as calcite, as a function of slip rate at constant (effective) normal stress. Temperature is assumed to remain at a fixed, ambient value in the Very‐LV and LV ranges (*Regimes 1–3*), with frictional self‐heating occurring in the HV region (*Regimes 4* and *5* at *V* > 1 mm/s). The CNS model predicts that an increase in applied temperature or in effective normal stress will shift the profile in *Regimes 1–3* to a higher velocity, and the profile in *Regimes 4* and *5* to a slightly lower velocity. This means that some of the transitions seen in Figure 4 might be absent or barely observed in some materials or at particular conditions. In the case of carbonate faults, only *Regimes 3–5* are observed in laboratory friction experiments conducted at room temperature (i.e., with no external heating) and at the slip rates and normal stresses typically employed—compare Figure 1 with Figure 4.

## 4 Extension of the CNS Model to High Velocities and Temperatures

To apply the CNS model to explain HVF, we will incorporate the evolution of porosity (Section 4.1) and grain‐size reduction (Section 4.2). Alongside the effects of increasing temperature caused by frictional heating, which we will describe using a continuum model in Section 5, these phenomena bring about the activation of multiple parallel creep mechanisms (Section 4.3).

### 4.1 Generalization to Include Grain‐Scale Creep and Effect of Evolving Porosity

Shearing of a dense granular gouge material, in which GBS by frictional or grain‐scale creep processes is sufficiently easy, leads to granular flow, whereby particles slide, roll, neighbor‐swap, and rearrange to produce both shear displacement and dilatant volumetric strain, that is, an increase in porosity (Budhu, 2010; Paterson, 1995). The presence of porosity during granular flow means that the local stresses on the grain contacts ($\tilde{\tau }\;\text{and}\;{\tilde{\sigma }}_{n}$) are higher than the macroscopically applied stresses ($\tau \;\text{and}\;\sigma_{n}$, Figure 3a). To account for the effect of porosity in accelerating grain‐scale creep via stress concentration at grain contacts, a suitable porosity function *f*(φ) must be introduced into the general creep law given in Equation 1b. This yields the following equations for shear and normal (compaction) strain rates by thermally activated deformation mechanisms (cf., Equation 1b, see also J. Chen et al., 2020):

(6a) $${\dot{\gamma }}_{pl}\left(\tau ,\varphi ,d,T\right)=A_{t}\;\text{exp}\left(-\frac{E_{a}}{RT}\right)\frac{{\left[\tau f_{t}\left(\varphi \right)\right]}^{n}}{d^{m}}.$$

(6b) $${\dot{\varepsilon }}_{pl}\left(\varphi ,d,T\right)=A_{n}\;\text{exp}\left(-\frac{E_{a}}{RT}\right)\frac{{\left[\sigma_{n}f_{n}\left(\varphi \right)\right]}^{n}}{d^{m}}\;.$$

Here, we assume that the same creep mechanism applies in both the shear and normal directions but that the creep properties of the gouge are anisotropic due to the anisotropic structure of the gouge, as idealized in the grain packing geometry assumed in Figure 3. Parameters *A*_*t*_ and *A*_*n*_ are preexponential factors containing geometric terms that account for this creep anisotropy in the shear and normal directions, respectively. The remaining parameters, that is, *E*_*a*_, *m*, and *n*, are the same in both directions and consistent with those appearing in the conventional creep law (Paterson & Olgaard, 2000). As addressed in the SI: Test S2, for a calcite gouge undergoing GBS with diffusion accommodation (Schmid et al., 1977), we take *A*_*n*_ ∼ *A*, where *A* is the preexponential constant in the classical creep law. The two geometrical factors can be related via a parameter,

(7) $$A_{t}/A_{n}=\epsilon .$$

For linear diffusion creep, ϵ can be approximated by the maximum value of tanψ (ϵ∼4.4, Text S2). As illustrated later by the simulation results, GBS with other nonlinear accommodation processes such as dislocation creep (*n* > 1) provides a negligible contribution to deformation (<<1%), therefore for simplicity we applied the same ϵ‐value to all the creep laws employed.

The porosity function in Equation 6a can be generally approximated as (Spiers et al., 2004)

(8a) $$f_{t}\left(\varphi \right)={\left(1-\frac{\varphi }{\varphi_{c}}\right)}^{-p}.$$

Here, *p* is grain‐packing‐dependent sensitivity factor that accounts for the changes in contact area (hence contact stress magnitude) and the length of the diffusion path (if a mass transfer process is involved), with changing porosity. As derived for IPS creep, it has a value of 1 if the creep rate is controlled by interfacial dissolution/precipitation and becomes 2 if controlled by diffusion (Spiers et al., 2004). Here, we apply this concept to a generalized creep mechanism (Equations 6a and 6b). Moreover, to avoid negative porosity in numerical computation, the porosity function is modified in the normal direction (J. Chen & Niemeijer, 2017) to

(8b) $$f_{n}\left(\varphi \right)={\left(\frac{\varphi_{c}-\varphi }{\varphi -\varphi_{0}}\right)}^{-p}.$$

Here, *φ*_0_ is the limiting porosity (2%), below which compaction cannot occur (while shear creep can). Such a limit in porosity has been observed in both natural and experimental faults after seismic slip (e.g., Smith et al., 2013) and likely corresponds to the (percolation) threshold for an interconnected pore network (Bryant & Blunt, 1992).

### 4.2 Incorporation of Grain‐Size Reduction

The original CNS model considers frictional behavior at a constant grain size and shear zone thickness, thus ignoring processes such as (de‐)localization and grain comminution. However, microstructural observations of samples recovered from high‐velocity experiments stopped at different shear displacements show that just before the onset of dynamic weakening, the accelerating slip has always (self‐)localized into a narrow shear band defined by a remarkable reduction in grain sizes from (sub)microns to nanometers (e.g., Demurtas, Smith, Prior, Spagnuolo, et al., 2019; De Paola et al., 2015; Smith et al., 2015). Following the strength‐velocity profile observed, this process corresponds to the strengthening phase prior to the dynamic weakening at *V* around 0.1 m/s (Figure 1). The underpinning physical process that forms these nanograins is not clear and deserves future study (De Paola et al., 2011; Reches & Dewers, 2005; Siman‐Tov et al., 2013; Smith et al., 2015). To capture the observations reported, we applied the following function to allow nominal grain size in the PSZ (*d*) to decrease from *d*_0_ to *d*_1_ as *V* accelerates to ∼0.1 m/s:

(9) $$d(V)=d_{0}+\left(d_{1}-d_{0}\right)\left[\frac{1}{2}+\frac{1}{2}\;\text{erf}\left(\frac{\text{log}\;V-\text{log}(0.1)}{\Delta }\right)\right].$$

A rate dependence of grain size is predicted by the grain comminution model proposed by Sammis and Ben‐Zion (2008) in which the grinding limit (finest attainable grain size) decreases with increasing strain rate. Assuming that a certain fraction of frictional slip energy goes to produce macroscopic cracks (Marone et al., 1990), the rate of grain crack production (or grain‐size reduction) has also be linked to the shear strain rate (Sleep, 1995). Adopting an error function and with *Δ* = 0.25, Equation 9 yields a fast, smooth change in grain size from *d*_0_ to *d*_1_ over a tenfold increase in velocity, that is, from 0.03 to 0.3 m/s, roughly consistent with experimental observations For the bulk zone where deformation is limited, we assume that the grain size does not reduce (by setting *d*_1_ = *d*_0_). As shown in our later parametric analyses, the overall picture does not change if grain size is not allowed to evolve, though if constant grain size of µm order is assumed then the dynamic weakening curve shifts to a higher temperature and velocity.

### 4.3 Incorporation of Multiple Parallel Creep Mechanisms

Since porosity (*φ*), grain size (*d*), and temperature (*T*) are continuously evolving during an HVF experiment, multiple creep mechanisms can operate within a deforming gouge layer, that is, in both the shear band and the bulk layer, with their relative importance varying continuously too. Here, we implement high‐temperature (nonfrictional) GBS with accommodation by diffusion and dislocation creep as well as low‐temperature IPS to be the candidate (contact) creep mechanisms. These mechanisms are treated as parallel processes in both normal and shear directions and automatically become dominant as τ, *φ*, *d*, and *T* change. Therefore, the total, purely plastic strain rates appearing in Equation 5 can be computed as the algebraic sum of the individual grain‐scale creep mechanism (*i*), that is,

(10a) $${\dot{\gamma }}_{pl}=\sum {\dot{\gamma }}_{pl}^{i}\left(\tau ,\varphi ,d,T\right)$$

and

(10b) $${\dot{\varepsilon }}_{pl}=\sum {\dot{\varepsilon }}_{pl}^{i}\left(\sigma_{n},\varphi ,d,T\right),$$

in the shear and normal directions, respectively. These creep processes will cause compaction of the gouge porosity (if present) that is mediated by dilatant, frictional granular flow. Further accounting for the spatial distribution of *T* over the bulk layer, the rate of sample deformation, as originally given in Equation 5, can be rewritten as

(11) $$V=W_{sb}{\left({\dot{\gamma }}_{gr}+\sum {\dot{\gamma }}_{pl}^{i}\right)}_{sb}+{\left(\overset{W_{bulk}}{\underset{0}{\int }}\sum {\dot{\gamma }}_{pl}^{i}dx\right)}_{bulk}.$$

Here, *x* is cross‐fault distance. Note that the relations given in both Sections 4.2 and 4.3 play a role only if *V* is above ∼1 mm/s, where grain‐size reduction and frictional heating play a role. At lower velocities, the model reduces to the original one that deals with fixed grain size and where IPS is the only active creep mechanism.

## 5 Application to HVF Experiments

We will now implement the above relations into a continuum modeling framework, to calculate the evolving temperature and friction profile across a bulk gouge layer plus marginal shear band (Figure 3a). The objective is to compute the dependence of steady‐state gouge layer friction on shearing velocity (cf., Figure 1), as well as the evolution of friction with displacement under fixed imposed conditions (cf., Figure 2).

### 5.1 Temperature Evolution due to Frictional Heating

During a HVF experiment, temperature rises due to heat production equal to the mechanical work dissipation given by ∫τVdt. For simulating the evolving or transient frictional behavior, we set up a finite element model (FEM) to calculate the temperature evolution across a planar gouge zone using the heat diffusion equation,

(12) $$\rho c\frac{\partial T}{\partial t}=k\frac{\partial^{2}T}{\partial x^{2}}$$

For simplicity, we employed a one‐dimensional (1‐D), across‐fault model, where *x* is position measured normal to the fault zone base. The model geometry considers a sample assembly that is commonly employed in HFV experiments, consisting of a gouge layer, two cylinders of the host rock as driving blocks, and two loading columns at both sides. Each component has its own dimensions and thermal properties (*ρ*, *c*, and *k*). A localized shear band was assumed to be located at one of the boundaries of the gouge layer. In this band, uniform grain size and homogeneous deformation were usually observed (Pozzi et al., 2018). To compute the average strain rates in this band, the average temperature (${\bar{T}}_{sb}$) was obtained using the relation: ${\bar{T}}_{sb}=\frac{1}{W_{sb}}\overset{W_{sb}}{\underset{0}{\int }}Tdx$. The heat source was specified in terms of an internal boundary condition at the center of the shear band given q=τV, which, using the above expression for ${\bar{T}}_{sb}$, is identical to using a width‐dependent heat source added to Equation 12. Beyond the shear band, the 1‐D temperature field calculated using the FEM was used to calculate creep strain rates as a function of *x* measured from the shear band center (Equations 6a and 6b). Note that we have tried using a 2‐D axisymmetric fault model to simulate laboratory fault, but the overall result obtained is the same as using the 1‐D model. To avoid complexity, we prefer to retain the 1‐D model in the present work.

Temperature of a shearing gouge would be continuously increasing during an HFV experiment, therefore, strictly speaking, there is no steady‐state temperature for a given velocity and normal stress, even during the nominal mechanical steady state (e.g., Di Toro et al., 2011). To compute the (nominal) steady‐state friction coefficient as a function of slip rate (cf., Figure 1), following the previous studies (e.g., Aharonov & Scholz, 2018), we assumed a characteristic temperature (*T*_*eq*_) corresponding to the nominal steady state of a shearing fault gouge at the given sliding velocity, which can be evaluated at the thermal‐weakening slip distance *D*_*th*_ (defined by Di Toro et al., 2011),

(13) $$T_{eq}=T_{0}+\frac{\tau }{\rho c}\sqrt{\frac{VD_{th}}{\pi \alpha }}.$$

In this equation, *T*_0_ is the initial or ambient temperature, *τ* is shear stress, *V* is sample shearing velocity which is equal to the loading velocity at steady state, and *ρ* and *c* are density and specific heat capacity of the fault gouge, respectively. Further, *α* is thermal diffusivity given as *α* = *k*/(*ρc*), where *k* is thermal conductivity. Di Toro et al. also found that *D*_*th*_ depends on the applied normal stress (*σ*_*n*_) following a power law equation:

(14) $$D_{th}=D_{0}{\sigma_{n}}^{-q},$$

where *D*_0_ and *q* depend on the material. As addressed above, at *D*_*th*_, the temperature may still be significantly lower than the indicative (maximum) value as experienced by the sample during the experiment.

### 5.2 Parameter Settings Characterizing Creep Mechanisms, Microstructure, and Thermal Structure

For the high‐T creep, following De Paola et al. (2015), we took the grain‐size‐sensitive flow law from *Regime* 3 of Schmid et al. (1977) to represent GBS accommodated by diffusion creep. Previous studies also showed that the bulk layer and occasionally the PSZ may have experienced dislocation creep (Demurtas, Smith, Prior, Spagnuolo, et al., 2019; Pozzi et al., 2018; Smith et al., 2013), for which we took the grain‐size‐insensitive, dislocation creep law from *Regime* 2 of Schmid et al. (1980). The related parameter values, including *A*, *E*_*a*_, *n*, and *m*, are given in Table 1. Note that these classic flow laws were determined from axisymmetric compression tests at HPT conditions. The way to translating these laws into Equations 6a and 6b, specifically, to resolve the preexponential constants *A*_*t*_ and *A*_*n*_, is detailed in SI: Text S2.

**Table 1:** *Parameters for the Creep Laws of Carbonate Used in This Study*

Mechanism^a^	*A* (s^−1^ μm^‐m^ MPa^−*n*^)	*E*_*a*_ (kJ/mol)	*n*	*m*	Source
GSS	4.79E6	213	1.7	3	Schmid et al. (1977), Regime 3
GSI	1.26E3	420	7.6	–	Schmid et al. (1980), Regime 2
IPS^b^			1	3	J. Chen and Spiers (2016)

^a^ GSI and GSS denote grain‐size‐insensitive and grain‐size‐sensitive creep, respectively.

^b^ IPS represent intergranular pressure solution, which, for a diffusion control process under uniform compaction, can be expressed as $\dot{\varepsilon }=B\frac{DCS\Omega }{RT}\frac{\sigma_{n}}{d^{3}}f\left(\varphi \right)$. Here, *B* is a geometric constant, *D* is the diffusivity of the dissolved solid within the grain boundary fluid (m^2^/s), *C* is the solubility of the solid in the fluid (m^3^/m^3^), *S* is the mean thickness of the grain boundary fluid phase (m), and Ω is the molar volume of the solid phase (m^3^/mol). Values of these parameters simply follow J. Chen and Spiers (2016).

For plastic deformation at low T, we assumed that the creep rate is controlled by a diffusion process (IPS), characterized by *n* = 1 and *m* = 3 (Table 1). The corresponding creep equation followed that used in our previous study (J. Chen & Spiers, 2016). Since the temperature dependence of the key parameter (i.e., solubility *C*) is only constrained up to 90°C (Plummer & Busenberg, 1982), we used constant kinetic parameters corresponding to ambient temperature (20°C). We note in the first place that the kinetics of IPS are extremely low at room temperature that it therefore plays a role only at very slow slip rates.

For the GB friction process, we assumed a reference GB coefficient of friction (${\tilde{\mu }}^{*}$) of 0.6 at 1 μm/s (J. Chen & Spiers, 2016). As the direct rate‐dependent parameter (i.e., *a*‐value) in the classical RSF model, the strain‐rate sensitivity parameter $a_{\tilde{\mu }}$ decreases with increasing *H*. Noda (2008) has illustrated that including a temperature dependence on the *a*‐value would cause a moderate weakening at velocities from 1 mm/s to 0.1 m/s. However, since a *V*‐strengthening regime is observed in this velocity range for carbonate rocks, we used a constant $a_{\tilde{\mu }}$‐value of 0.01, which allows capturing the overall velocity dependence of steady‐state friction at low velocities (<1 mm/s, Figure 1).

To represent the typical microstructure observed in experiments, we considered a 20‐μm‐thick PSZ developed at one margin of an 800‐μm‐thick gouge layer (e.g., De Paola et al., 2015; Pozzi et al., 2018), except where otherwise stated. For both zones, we assumed an initial porosity of 18%, and initial grain sizes were set to be uniform at 1 and 5 μm, respectively. As *V* approaches ∼0.1 m/s, the grain size in the PSZ was forced to decrease from *d*_0_ (=1 μm) to *d*_1_ (=10–100 nm) following Equation 9, which contains a *Δ*‐value of 0.25 to specify the rate of decrease (Table 2).

**Table 2:** *Reference Constitutive Parameters Adopted in This Modeling*

Para.	Definition	Value or function	Source or note
*Material properties for a calcite fault gouge*			
*H*	Geometry constant	0.57	J. Chen and Spiers (2016)
$a_{\tilde{\mu }}$	Rate dependence of GB friction	0.01	This study
${\tilde{\mu }}^{*}$	Reference GB friction at 1 μm/s	0.6	J. Chen and Spiers (2016)
*φ*_0_	Limited porosity	2%	J. Chen and Niemeijer (2017)
*φ*_*c*_	Critical porosity	20%	J. Chen and Spiers (2016)
*p*	Sensitive factor to porosity	2	J. Chen and Niemeijer (2017)
*Parameters for a simulated carbonate fault in HVF experiments*			
$\sigma_{n}$	Effective normal stress	8.5–25 MPa	Pozzi et al. (2018)
*T*_0_	Ambient temperature	20°C	
*W*_*sb*_	Shear band thickness	20 μm	Pozzi et al. (2018)
*W*	Gouge layer thickness	1.0–3.0 μm	Smith et al. (2015)
*d*_0_	Mean grain size of PSZ at low *V*	1 μm	J. Chen and Spiers (2016)
*d*_1_	Grain size of PSZ at *V* > 0.1 m/s	10–100 nm	Smith et al. (2015), De Paola et al. (2015), and Green et al. (2015)
*d*_*bulk*_	Mean grain size in the bulk layer	5 μm	De Paola et al. (2015)
*φ*_*i*_	Initial porosity at low *V*	0.18	J. Chen and Spiers (2016)
*K*	Fault stiffness	300 GPa/m	This study
*V*_*imp*_	Imposed slip rate	Velocity profile	Mimicking experiments
*D*_*th*_	Thermal equilibration distance $D_{th}=D_{0}{\sigma_{n}}^{-q}$	*D*_0_ = 10 m; *q* = 1	For nominal steady state
*ρ*	Density of gouge	2,700 kg m^−3^	X. Chen et al. (2013)
	Density of steel	8,000 kg m^−3^	Cverna (2002)
*c*	Specific heat capacity of gouge	800 J kg^−1^ K^−1^	X. Chen et al. (2013)
	Specific heat capacity of host block	500 J kg^−1^ K^‐1^	Cverna (2002)
*k*	Thermal conductivity of gouge	2.16 W m^−1^ K^−1^	X. Chen et al. (2013)
	Thermal conductivity of host block	15 W m^−1^ K^−1^	Cverna (2002)

For simulating the transient behavior during a HVF experiment, we constructed a fault structure based on the sample assembly used in the experiment. We assumed that the gouge layer is sandwiched between two 50 mm‐long host blocks made of either stainless steel or Ti‐alloy depending on the experiment to be simulated, and further between a pair of 50 mm‐long loading column made of stainless steel. The densities (*ρ*) and thermal properties (*k* and *c*) of the different components (i.e., gouge and host rock) are given in Table 2. We set the ambient temperature *T*_0_ = 20°C and normal stress to be the applied in the simulated experiments.

For predicting the nominal steady state, we assumed *D*_0_ = 10 m and *q* = 1, predicting *D*_*th*_ = 0.57 m at *σ*_*n*_ of 18 MPa, which is consistent with the slip‐weakening curve observed in the experiment (Figure 2). For the computation of steady state, the temperature and thus deformation rate is not specified in the bulk layer and therefore neglected. This is reasonable since, as verified by the simulation results of transient behavior, the bulk layer has a negligible contribution to the shear deformation (<1%).

### 5.3 Solution Method

The experimental fault is represented as a spring‐slider system composed of a linear spring of stiffness *K*, activated by the imposition of an evolving velocity at the load point (*V*_*imp*_, Figure 3a). In this study, only stable sliding behavior was simulated, therefore a relatively stiff fault was used (300 GPa/m). Neglecting inertia, the evolution of shear stress can be written in a time‐derivative form as

(15) $$\frac{\partial \tau }{\partial t}=K\left(V_{imp}-V\right)$$

This, combined with equations for the evolution of porosity (Equation 3a), constitutes two governing ordinary differential equations (ODEs) for the present model. To simulate the frictional curve in a typical HVF experiment, these ODEs, coupled with the grain‐size evolution (Equation 9) and a 1‐D FEM for temperature evolution (Equation 12), were solved simultaneously using the finite element package Comsol.

For a given velocity and normal stress, the steady‐state solution can be obtained by setting the time derivatives (∂τ/∂tand ∂φ/∂t) to be 0 and using the expressions of characteristic temperature upon reaching the nominal steady‐state friction (Equations 13 and 14).

### 5.4 Simulation Results

#### 5.4.1 Prediction of Steady‐State Friction at Low‐to‐High Velocities

**Figure 5:**
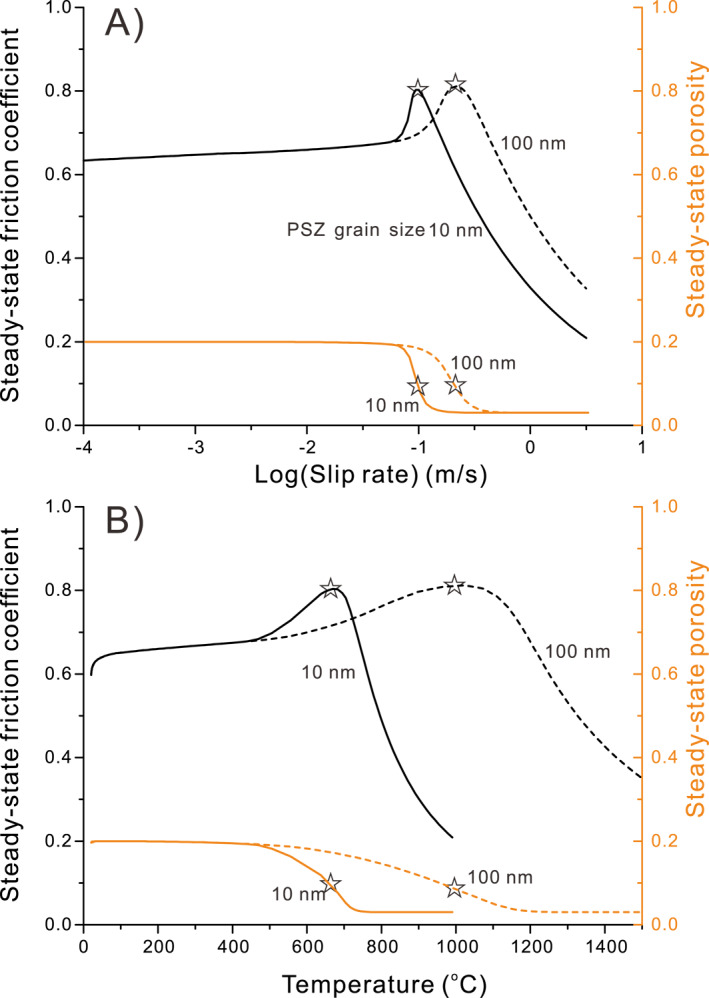
The steady‐state friction coefficient and PSZ porosity for a simulated carbonate fault as a function (a) slip rate and (b) temperature, predicted by the CNS model (cf., Figure 1). PSZ, principal slip zone; CNS, Chen–Niemeijer–Spiers.

**Figure 6:**
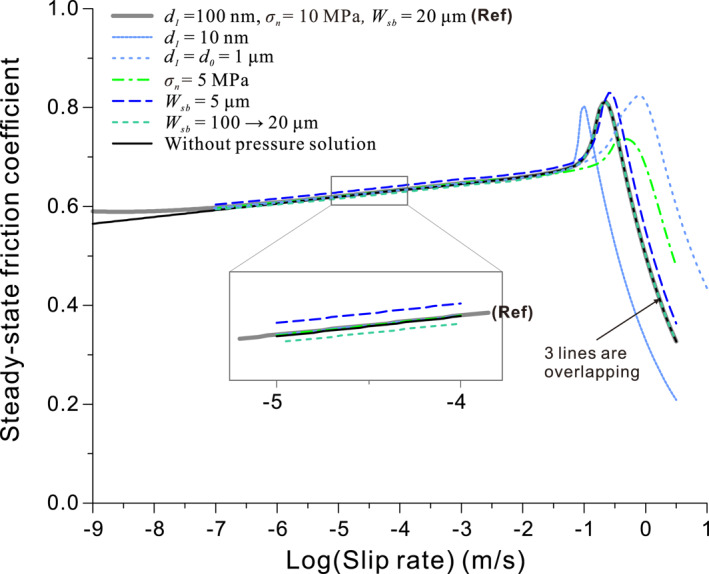
The sensitivity of the computed steady‐state friction to variations in the parameter values. The comparison was done with respect to a reference case; each curve changes one parameter. The reference and changed values are indicated in the legend.

For simulating the steady‐state behavior of the type obtained in experiments shown in Figure 1, we assumed a gouge zone with the internal structure as described above and we used a constant effective normal stress (*σ*_*n*_) of 10 MPa. In Figures 5a and 5b, the predicted apparent steady‐state friction coefficient (*μ* = *τ*/*σ*_*n*_) and shear band porosity are plotted against the logarithmic velocity and against temperature, respectively. To test the effect of grain size, we used 10 and 100 nm as the nominal grain size of nanoparticles generated at high *V* (>0.1 m/s). For both grain sizes, the results are generally consistent with the experimental observations (cf., Figures 1 and 5a): a gentle logarithmic *V*‐strengthening followed by a strong *V*‐strengthening, and then a sharp dynamic weakening, as *V* increases (the same results are given in Figure 6 showing a wider *V*‐range). The strong *V*‐strengthening occurs at a temperature of about 500°C, in association with the onset of porosity reduction (Figure 5b). Dynamic weakening is accompanied by ongoing reduction in porosity, while strength drops due to the effect of shear heating, that is, due to the onset of easy flow by GBS with diffusion accommodation. A larger grain size corresponds to a higher critical velocity and critical temperature for the onset of dynamic weakening (*V*_*w*_ and *T*_*w*_, as given by the stars in Figures 5a and 5b, respectively), that is, *V*_*w*_ = 0.2 m/s and *T*_*w*_ = 1,000°C, versus *V*_*w*_ = 0.1 m/s and *T*_*w*_ = 650°C, for *d*_1_ = 100 and 10 nm, respectively. Similar results could also be predicted by the model assuming fixed grain size (i.e., *d*_1_ = *d*_0_), but the *V*_*w*_ and *T*_*w*_ values predicted would be much higher (Figure 6).

Besides varying grain size, we performed sensitivity analyses on the normal stress (*σ*_*n*_) and PSZ thickness (*W*_*sb*_), with respect to a reference case. As shown in Figure 6, the computed results show that a lower *σ*_*n*_ corresponds to a higher velocity for the onset of dynamic weakening (*V*_*w*_), plus a lower but broader peak stress, while a thinner PSZ leads to a slightly higher level of friction at low velocities (see the inset) and a higher *V*_*w*_. Regarding the PSZ thickness, we also investigated the effect of progressive localization (i.e., from ∼100 to 3 μm, as reported for a carbonate gouge by Smith et al. [2015]), which was inferred to occur contemporaneously with the nanopowder formation. Similarly to Equation 9, this was simulated by letting *W*_*sb*_ evolve as a function of *V*, employing the following equation:

(16) $$W_{sb}(V)=W_{sb}^{0}+\left(W_{sb}^{1}-W_{sb}^{0}\right)\left[\frac{1}{2}+\frac{1}{2}\;\text{erf}\left(\frac{\text{log}\;V-\text{log}(0.1)}{\Delta }\right)\right].$$

Here, $W_{sb}^{0}$ and $W_{sb}^{1}$ are the initial and final thicknesses of the PSZ. The result showed that an evolving PSZ thickness does not change the result for *V* > 0.1 μm/s (cf., the reference case with constant thickness, $W_{sb}=W_{sb}^{1}$, Figure 6), which is not very surprising since the same thicknesses were used in this *V*‐range. However, a slightly lower friction is generated at low velocities due to the lowered shear strain rate (see the inset). In all of the above simulations, we included IPS in an adsorbed water film on grain boundaries as one of the creep mechanisms. However, omitting IPS does not change any of our results, except for *V* < 0.1 μm/s (Figure 6). From now on, we proceed to simulate the transient behavior during HVF experiments and therefore will not use this mechanism in our simulations.

#### 5.4.2 Prediction of Transient Behavior in HVF Experiments

We next proceeded to simulate the full friction versus displacement behavior recorded in the HVF experiments performed on dry crushed carbonate fault gouges by De Paola et al. (2015) and Smith et al. (2015). The experiments by Smith et al. also reported the measured evolution of gouge thickness. All these experiments were typically arrested at predetermined displacements, digitally controlled using an electric servomotor equipped with a signal function generator. The input *V*‐profiles in our simulations mimicked those imposed in the experiments (see details in SI: Text S3). For De Paola et al.'s experiments, we assumed a 0.8‐mm‐thick gouge layer and used *σ*_*n*_ of 12 and 18 MPa, while for Smith et al.'s runs we simulated a thicker layer of 2 mm and used *σ*_*n*_ of 8.5. MPa—following the experimental details described by these authors. Based on the microstructures reported in both of these studies, we assumed a PSZ grain size evolving from 1 μm to 20 nm. Both studies used stainless steel as the “host rock.” Parameters related to the creep laws and structure of the experimental fault are given in Tables 1 and 2.

**Figure 7:**
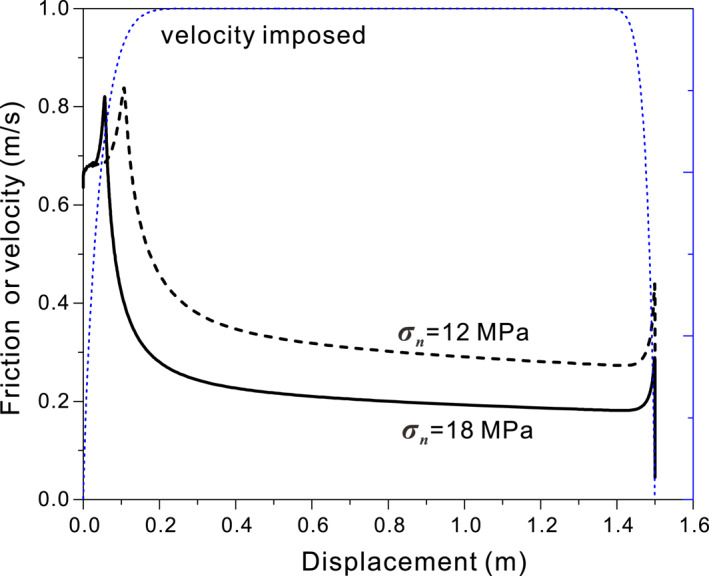
Reproducing the HVF experiments by De Paola et al. (2015), showing the evolution of friction coefficient with shear displacement under two normal stresses (cf., Figure 2). HVF, high‐velocity friction.

**Figure 8:**
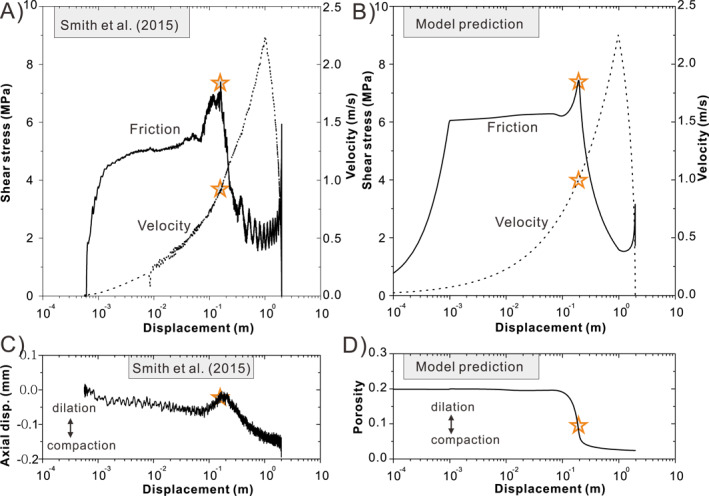
Prediction of the HVF experiment by Smith et al. (2015) at normal stress of 8.5 MPa. (a and b) Comparison between the experimental and predicted results for the evolution of friction against the logarithm of shear displacement. (c and d) Comparison between the experimental and predicted results for compaction/dilatation. HVF, high‐velocity friction.

As seen in the experimental results by De Paola et al. (2015), for both normal stresses (12 and 18 MPa) simulated, our model predicts curves of apparent friction coefficient versus displacement with an initial value of 0.7, followed by a strong transient strengthening to a peak value (*μ*_*pk*_) of ∼0.8, and then a sharp dynamic weakening to a nominal steady‐state friction (*μ*_*nss*_, Figure 7). Higher normal stress causes a shorter preweakening distance *u*_*w*_ (0.07 vs. 0.12 m) and a lower *μ*_*nss*_ (0.2 vs. 0.3 at *σ*_*n*_ of 18 and 12 MPa, respectively). As slip continues at constant *V*, *μ*_*nss*_ slightly decreases and is followed by an increase along with the deceleration. All these results are generally consistent with the friction–displacement curve seen in the experiment (cf., Figure 2 or Figure 1 of De Paola et al., 2015). For comparison with the experimental data as plotted by Smith et al. (2015), the predicted evolution of shear stress is plotted against the logarithm of slip distance in Figure 8. The result shows that dynamic weakening occurs at ∼0.1 m displacement and when *V* accelerates to ∼0.9 m/s (indicated by the stars), which is remarkably consistent with the experimental results (cf., Figures 8a and 8b). The model also predicts significant compaction in association with the transient strengthening and dynamic weakening seen in the experiments at velocities of 0.07–1.0 m/s. This correspondence is also broadly consistent with the axial displacement observed in the experiments (cf., Figures 8c and 8d), except that in Smith et al.'s experiment the strengthening phase was (sometimes) accompanied by minor dilatancy, while our model predicts continued compaction. We notice that our prediction agrees better with more recent results by Rempe et al. (2017), Pozzi et al. (2018), and Demurtas, Smith, Prior, Spagnuolo, et al. (2019). We infer that the dilatancy seen by Smith et al. was in part due to the use of thicker gouge layers (2–3 mm) and therefore a larger contribution from the bulk (outside the shear band). Such a contribution could be due to (elastic) coupling between normal and shear deformation so that increasing shear stress in the strengthening phase could cause dilatation.

**Figure 9:**
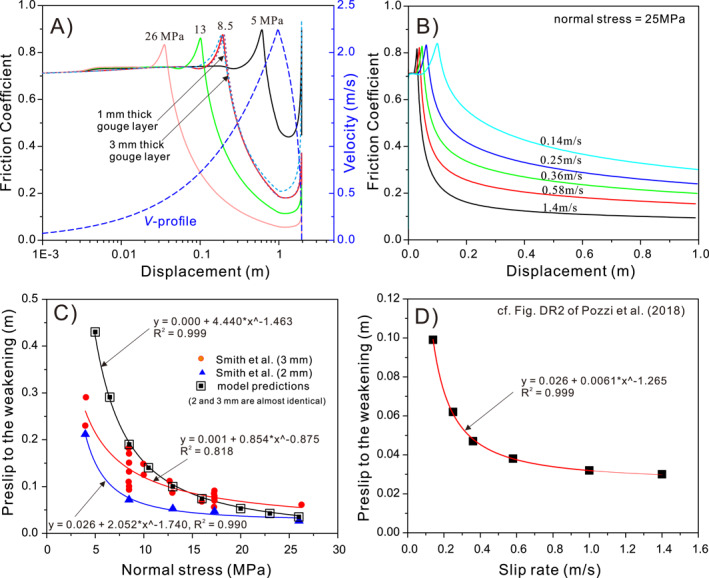
Simulation of the HVF experiments performed under (a) varied normal stresses by Smith et al. (2015) and (b) varied nominal slip rates by Pozzi et al. (2018). (c and d) The predicted preweakening slip distance as a function of normal stress and slip rate, respectively. In (c), note that the data reported by Smith et al. for gouge layer thicknesses of 2 and 3 mm are significantly different, whereas the corresponding simulation results show almost no effect of gouge thickness. HVF, high‐velocity friction.

We further investigated the effects of (effective) normal stress (*σ*_*n*_), nominal sliding velocity (*V*), and gouge layer thickness (*W*), following Smith et al. (2015) and Pozzi et al. (2018). Note that Pozzi et al. used Ti‐alloy as the “host rock,” with the other settings the same as De Paola et al. (2015). The modeling results show that a higher *σ*_*n*_ or *V* leads to a lower peak and nominal steady‐state friction (*μ*_*pk*_ and *μ*_*nss*_), as well as a shorter preweakening slip distance (*u*_*w*_, Figures 9a and 9b). The modeled dependence of *u*_*w*_ on *σ*_*n*_ can be best fitted by a power law assuming zero intercept ($\mu_{w}=a\times \sigma_{n}^{-b}$, Figure 9c), while that on *V* is best fit by the same power law with a positive intercept ($\mu_{w}=a\times V^{-b}+c$, Figure 9d), suggesting nearly constant *u*_*w*_ at high velocities (*V* > 1 m/s). These predicted trends are broadly consistent with those observed in experiments (Pozzi et al., 2018; Smith et al., 2015), though Smith et al. (2015) described the *u*_*w*_–*σ*_*n*_ relation using a logarithmic law. Considering the scatter of experimental data, we found that their data can be fitted with a power law equally well (Figure 9c). Our results also show that a thicker gouge layer leads to a longer preweakening distance *u*_*w*_ (see the dashed lines in Figure 9a), which is again consistent with the experimental results. However, the predicted variation in *u*_*w*_ for different thicknesses at fixed normal stress is very small (<5 mm between 2‐ and 3‐mm‐thick gouge layers) compared to the variation observed in the experiments (<40 mm, Figure 9c). We infer that this discrepancy in the variation in *u*_*w*_ is partially because our model assumes a mature microstructure in the first place (i.e., with the presence of a shear band, Figure 3) such that the thickness of the gouge layer only affects the thermal diffusion process and therefore has little effect on the frictional behavior, while in the experiments a larger displacement is required to develop such a microstructure and heat the temperature high enough to activate weakening in a thicker gouge layer.

Finally, to test the sensitivity of the computed friction versus displacement curves to the parameter values chosen, we performed a second parametric analysis on the key parameters, that is, shear band thickness (*W*_*sb*_), grain size (*d*_0_ or *d*_1_), threshold porosity (*φ*_0_), and the ratio between the two preexponential creep law constants (*A*_*t*_/*A*_*n*_). Varying these quantities in a physically reasonable range does not change the general picture of the predicted results. Indeed, similarly shaped frictional weakening curves are predicted (SI: Figure S3) but with a slight translation, and slightly different peak and nominal steady‐state friction (*μ*_*pk*_ and *μ*_*nss*_) as well as the preweakening distances (*u*_*w*_). Specifically, smaller *W*_*sb*_ or *A*_*t*_/*A*_*n*_, or larger *d*, correspond to higher values of *μ*_*pk*_, *μ*_*nss*_, and *u*_*w*_; decreasing *φ*_0_ also leads to higher values of *μ*_*pk*_ and *μ*_*nss*_ but does not affect *u*_*w*_ efficiently.

## 6 Discussion

### 6.1 General Features of the CNS Model Over the Full Velocity Range

In the present study, we have extended a previously proposed model (CNS model, J. Chen & Spiers, 2016) that accounts for the frictional behavior at the low‐velocity (*LV*) range, to high velocities (*HV*) by introducing multiple grain‐scale deformation mechanisms activated by frictional heating. As velocity increases, the model now predicts a continuous transition in the deformation mechanisms, from granular flow with partial accommodation by plasticity at low velocities and temperatures, to GBS with increasing accommodation by solid‐state diffusion at high velocities and temperatures. This extended CNS model, in combination with our earlier effort in bridging the very‐*LV* range (J. Chen, Niemeijer, & Spiers, 2017; J. Chen & Niemeijer, 2017; J. Chen et al., 2020), now covers the full spectrum of slip velocities from earthquake nucleation to seismic slip rates (Figure 4).

We have shown that the extended model can explain both the steady‐state and transient frictional behavior observed in HVF experiments on carbonate fault gouges at dry, room humidity conditions (Figures 1 and 2). In the following, we will compare the CNS model with previous models and illustrate the key ingredient(s) that bridges the *LV* and *HV* deformation regimes. We will also address questions related to extrapolation to nature and propose future studies.

### 6.2 Comparison With Previous Models: The Bridging Role of Porosity

**Figure 10:**
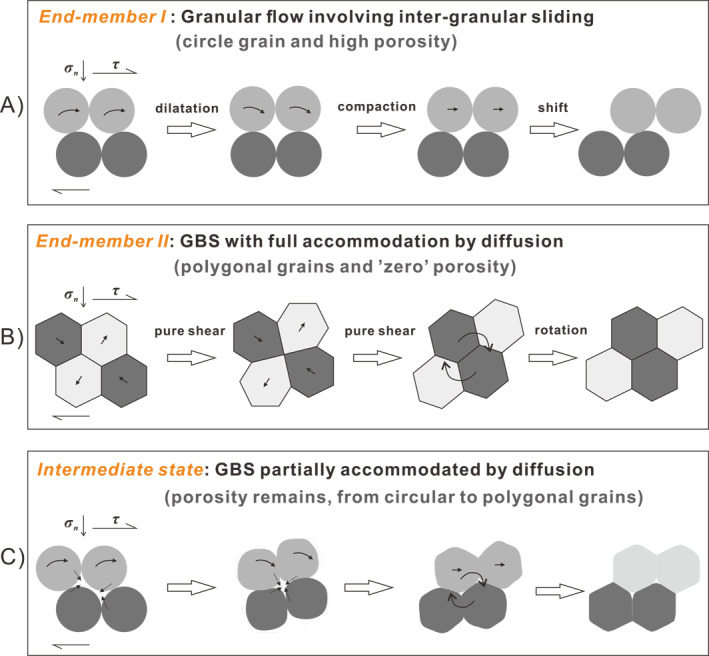
Schematic diagrams showing a unit strain deformation at high velocities where the dominant deformation mechanism undergoes a transition between two end‐members as velocity increases. Note that the average grain sizes are different for the two end‐members. See detailed explanation in the text and also refer Figure 3b.

The good performance of the CNS model in predicting the steady‐state and transient behaviors supports the conceptual model in Figure 3b for explaining carbonate friction at high velocities (*V* ≥ ∼1 mm/s). As an update, Figure 10 illustrates the grain‐scale deformation steps for the transition of deformation mechanisms, namely, from granular flow involving (frictional) intergranular sliding (Paterson, 1995) to high‐temperature GBS accommodated by diffusion creep (Ashby & Verrall, 1973). For facilitating the description, we assume that critical‐state granular flow achieved at *V*_*c*_ = 1 mm/s, the onset of dynamic weakening at *V*_*w*_ = 0.1 m/s, and complete weakening at *V* ≥ *V*_*f*_ = 1 m/s.

At *V* = *V*_*c*_ (*End‐member I*), the deformation will occur by critical‐state granular flow, with the macroscopic porosity asymptotically approaching the (maximum) critical value (Figure 10a) and with frictional sliding interactions between grains. At this point, the shear strength is purely attributed to grain boundary frictional sliding ($\mu =\tilde{\mu }$), as given by Equation 3b.

When *V* is greater than *V*_*c*_, a shearing fault gouge will start to generate high temperatures in addition to finer and finer nanoparticles being produced. At even higher velocities (*V* ≥ *V*_*f*_, *End‐member II*), and resulting high temperature and decreased grain size, GBS with accommodation by diffusion will become fast enough to fully accommodate the shear deformation, resulting in near‐zero porosity material with polygonal grains and low sensitivity of strength to normal stress (Figure 10b). At this point, shear strength will be controlled by solid‐state, grain boundary diffusion—that is, Equation 6a, where *n* = 1 and *m* = 3, or by Equation 1b as φ reduces to 0.

At intermediate *V* (*V*_*c*_ < *V* < *V*_*f*_), deformation will be achieved by a mixture of these two end‐member processes. Grains within the PSZ will attain some state between the two end‐members, that is, with intermediate porosities and grain shapes (Figure 10c). As *V* increases in this range, the shear strength will first increase markedly due to compaction (i.e., through the increase of tanψ in Equation 3b) and then weaken rapidly as the slip zone becomes hotter and diffusive mass transfer becomes faster. By analogy with the frictional healing effect caused by compaction achieved during a pause in slip, that is, during the hold period of a slide‐hold‐slide experiment, Equation 17b of J. Chen et al. (2020) can be used to estimate the maximum strength gained due to compaction in the interval *V*_*c*_ < *V* < *V*_*w*_, that is, prior to the onset of weakening at *V ≈ V*_*w*_:

(17) $$\Delta \mu_{gain}\leq 3.5H\left(\varphi_{c}-\varphi \right).$$

where φ is the instantaneous porosity at the peak strength. The application of this relation here is verified by our steady‐state simulation of Figure 5. Those results show that the gouge compacted by ∼10% while the friction coefficient increased by 0.17 to attain the peak strength—which is roughly consistent with the estimate $\Delta \mu_{gain}\leq 0.199$ made from Equation 17.

The *End‐member II* process is similar to the classical superplasticity model proposed by Ashby and Verrall (1973) for material under “pure shear” deformation (hereafter referred to as the *A&V model*). The only difference is that during a simple shear test, a dense gouge undergoes “pure shear” deformation plus nondissipative rigid rotation (Figure 10b). Combined operation of GBS plus diffusion has also been previously addressed by Raj and Ashby (1971), but these authors assumed zero (negligible) resistance to GBS. Conventionally, this is true since GBS, which is usually believed to be the main mechanism underlying superplastic flow at high temperature, is a nonfrictional process involving easy motion of grain boundary dislocations with no effect of normal stress. It is only associated with dilatation (i.e., the creation of porosity against normal stress) when other mechanisms such as diffusion are too slow to fill in the growing cavities, as in the simulated case at *V* < *V*_*w*_ in the present model.

We posit that porosity creation (dilatation) and elimination (compaction) are the keys in controlling gouge behavior across the full range of velocity, and hence in controlling both LV and HV slip, at least for carbonate friction at room‐dry conditions. It is the compaction creep in the PSZ that is responsible for the strong *V*‐strengthening in the steady‐state profile. Such *V‐*strengthening behavior prior to dynamic weakening (*V*_*c*_ < *V* < *V*_*w*_) has also been observed in other materials such as halite, glass beads, serpentine, granite, and phyllosilicate‐rich gouges (e.g., Boneh & Reches, 2018; J. Chen, Niemeijer, & Spiers, 2017; J. Chen & Niemeijer, 2017; Ferri et al., 2011; Kohli et al., 2011; Kuwano et al., 2013; Reches & Lockner, 2010; see a review in Bar‐Sinai et al., 2014). Although the specific dynamic weakening mechanisms may be completely different in different materials, most likely they are all of a thermal origin (Rice, 2006). As *V* or *T* increases and grain size is reduced (*V < V*_*w*_), any thermally activated deformation process can cause compaction of a shearing granular fault gouge—or indentation of asperities into sliding bare rock surfaces. These will increase the average dilatancy angle and hence frictional strength before thermally activated weakening become efficient enough to dominate shear strength and cause wholesale strength loss (J. Chen, Niemeijer, & Spiers, 2017). High‐resolution measurements of compaction within the PSZ are required to check this hypothesis in future experiments. For a natural fault to have dynamic weakening, this strength needs to be overcome before *V* can accelerate to *V*_*w*_, as discussed in the following.

### 6.3 Implications for Rupture on Natural Carbonate‐Hosted Faults at Shallow Depths

In experimental faults filled with calcite‐dominated gouge and sheared at rupture nucleation velocities (<10 µm/s), a transition from *V*‐strengthening to *V*‐weakening occurs at temperatures of ∼80°C (J. Chen et al., 2015; Verberne, Plümper, et al., 2014), which is consistent with the upper bound in aftershock depths following the 2009 Mw 6.3 L'Aquila earthquake, for example (∼3 km, Chiaraluce, 2012; Valoroso et al., 2013). At shallow depths (<3 km), in addition to the stabilizing effect of *V*‐strengthening at low velocities (*V* < 1 mm/s), the marked *V*‐strengthening seen prior to dynamic weakening in experiments at velocities *V* in the range (1 mm/s > *V* > 0.1 mm/s, Figure 1)—and predicted by the CNS model (Figures 5 and 6)—will delay or tend to inhibit the occurrence of dynamic weakening. Our model results show that this strengthening effect decreases in magnitude at higher normal stress or deeper crustal levels (Figures 9a and 9c) and that overcoming this barrier, at any given normal stress, requires dramatically larger displacement if the fault is sliding at a lower slip rate (<0.5 m/s Figures 9b and 9d). Such an impeding effect would imply complex spatial distributions and modes of coseismic and postseismic slip in carbonate terrains. A direct implication is that a seismically impeded rupture would undergo less coseismic slip but larger afterslip due to the high shear stress associated with the impeding effect. This hypothesis can be tested in the future by numerical modeling of rupture propagation in a continuum modeling framework. Interestingly, in the Central and Northern Apennines of Italy, the destructive seismic rupture zones coexist with slow aseismic rupture phenomena (Amoruso et al., 2002; Crescentini et al., 1999).

### 6.4 Future Developments

In this study, we have applied an extended form of the CNS model to simulate experiments in a continuum model framework, allowing for frictional heating, grain‐size reduction, as well as additional grain‐scale creep mechanisms to be incorporated, resulting in the activation of dynamic weakening at seismic slip rates. However, experiments suggested that dynamic weakening occurs in association with the spontaneous development of microslip zones (i.e., slip localization) and the velocity‐dependent creation of nanoparticles (De Paola et al., 2015; Smith et al., 2015). Self‐localization suggests that hypotheses such as competition between *V*‐strengthening friction and any strain weakening mechanism need to be considered, as both are strain rate dependent but in the opposite sense (Rice et al., 2014) or that more advanced theories such as the Cosserat continuum approach need to be applied to address the effects of grain‐size reduction (Sulem et al., 2011). Hypotheses for the formation of nanograins in carbonate faults include thermal decomposition (Han et al., 2007), dynamic pulverization (Reches & Dewers, 2005), dynamic milling (Siman‐Tov et al., 2015), migration of fast‐moving dislocation by stress shocking (Spagnuolo et al., 2015), and amorphization followed recrystallization (Ohl et al., 2020). During “postseismic” slip, the nanograins in the PSZ will tend to grow in size and heal the fault (Green et al., 2015; Pozzi et al., 2018), which would in turn affect the interseismic behavior of the fault. An ideal model in the future should capture some of these effects (Barbot, 2019; Sleep et al., 2000).

The extension of the CNS model in the present study is limited to carbonate fault rocks, which favor the thermal (dynamic) weakening at high temperature, due to the onset of diffusion‐accommodated GBS or superplastic flow. Other weakening mechanisms such as flash heating, decarbonation, and thermal pressurization (when water is present) may also play some role at different stages of a seismic slip event. Future work needs to integrate the magnitude of these effects and to integrate them into the model where significant.

As indicated in Section 6.2, it would be interesting to apply the CNS model to explore the modes of fault rupture at shallow depths of a carbonate fault zone. It would be more interesting to investigate the spontaneous fault behavior at seismogenic depths (i.e., ≥4 km), where a calcite gouge shows *V*‐weakening behavior so that fault instability can occur spontaneously, generating rupture that can then propagate to shallow depths. As the rupture propagates, whether fault slip is fast or slow depends on whether the acceleration can overcome the two types of barriers. To examine these processes, numerical simulations in a continuum model framework are planned in the future by implementing the present model into an earthquake cycle simulator (Lapusta & Rice, 2003; Noda et al., 2009; van den Ende et al., 2018, 2020). Finally, the present work only explored the fault behavior of carbonate rocks under nominally dry conditions. One important effort would be to apply the model to other materials and consider the coupling with pore fluid (pressure), under future developments to the continuum model framework.

## 7 Conclusions

Previous HVF experiments on carbonate fault rocks at nominally dry conditions have been used to propose that dynamic frictional weakening is due to the operation of GBS with diffusion accommodation within a PSZ consisting of nanocrystalline calcite (De Paola et al., 2015; Green et al., 2015; Smith et al., 2015). In the present study, we incorporated this mechanism into a microphysical model (CNS model) originally developed for modeling the frictional behavior of granular fault gouges at low velocities (<1 mm/s). In the application of the model to typical HVF experiments (≥1 mm/s), we used a 1‐D continuum modeling framework to account for temperature evolution in and around the PSZ.

The combined microphysical and continuum models predict results that capture all of the main features and trends seen in HVF experiments, including both steady‐state and transient aspects, such as the evolution of friction coefficient with shear displacement, with the reasonable quantitative agreement. Notably, the model (1) predicts steady‐state friction versus velocity over the full spectrum of velocities involved in the seismic cycle, from earthquake nucleation to seismic velocity; (2) reproduces typical HVF experiments, including both the evolution of friction and porosity; and (3) predicts dynamic weakening after a marked transient strengthening phase, which shortens with increasing normal stress and velocity, as seen in experiments. We propose that the generation of porosity through dilatant granular flow of gouge, and its collapse due to the onset of thermally activated compaction creep, with increasing temperature, is the key ingredient in explaining the evolution of shear strength of a rapidly accelerating fault containing calcite gouge. Since the model is based on realistic fault structure and microphysical deformation processes, it can thus provide an improved basis for extrapolating lab‐derived friction data to natural fault conditions. Future work will consider an implementation of the extended model into earthquake cycle simulations in a continuum framework.

## Supporting information

Supporting Information S1

## Data Availability

The work presented here is theoretical and no data have been produced requiring online publication.
