# Supplementary material for: Microphysical Modeling of Carbonate Fault Friction at Slip Rates Spanning the Full Seismic Cycle
Source: J Geophys Res Solid Earth. 2021 Mar 25;126(3):e2020JB021024. doi: 10.1029/2020JB021024 (PMC8047899; doi:10.1029/2020JB021024)
Supplement: Supplementary file 1 — Supporting Information S1 [file JGRB-126-e2020JB021024-s001.pdf]

AGU PUBLICATIONS

Journal of Geophysical Research: Solid Earth

Supporting Information for

Microphysical modeling of carbonate fault friction at slip rates spanning the full seismic cycle

Jianye Chen[1, 2, 3*], A. R. Niemeijer[2], Christopher J. Spiers[2]

[1] *State Key Laboratory of Earthquake Dynamics, Institute of Geology, China Earthquake Administration, 100029, Beijing, China.*

[2] *HPT Laboratory, Department of Earth Sciences, Utrecht University, Princetonlaan 4, 3584 CB Utrecht, The Netherlands*

[3] *Now at Geoscience & Engineering Department, Delft University of Technology, Stevinweg 1, 2628 CN Delft, The Netherlands*

[*] Corresponding author: Jianye Chen (e-mail: j.chen3@uu.nl)

Address: Princetonlaan 4, 3584 CB Utrecht, The Netherlands

Content

Text S1; Figure S1;

Text S2; Figure S2;

Text S3; Figure S3.

Introduction

Text S1 decribes the main results of Figure S1, which is the same as Figure 1, but plotted steady-state friction coefficeint against power density. Text S2 and Figure S2 address the translation of parameters from a conventional creep law to the proposed creep law in this study for a simple shear experiment (Eqs. 6a and 6b). Text S3 explains the velocity profile used for simulating the high-velocity friction (HVF) experiments. Figure S3 presents the simulation results of friction vs. displacement curve from the parametric analyses.

Text S1: Steady-state Friction as a Function of Power Density

In Figure 1, the dataset at the high-velocity (LV) range ($V > 0.1$ m/s) tend to show some normal stress dependence that the steady-state friction coefficient is lower at higher normal stress. Following the previous study (Di Toro et al., 2011), we have compiled the same dataset, plotting steady-state friction as a function of power density (PD = shear stress times slip velocity), which basically reflects the input of mechanical energy into the fault system (Figure S1). A

similar graph has been previously plotted for carbonate fault materials by Boneh et al. (2013), but with a smaller number of data. In Boneh et al.'s graph (their Figure 5), the normal stress and velocity dependences of steady-state friction can be merged into a single 'master curve' (see also Di Toro et al. (2010) for other materials). With increased number of studies, our new graph shows that the data scattering seen in Figure 1 can be narrowed to some extent, but still does not improve the agreement between different data sets, i.e. shows no better tendency for the data to converge towards a single "master curve". Interestingly, we found that the values of steady-state friction given by Han et al. (2011) and Violay et al (2013) are lower than other studies at a given powder density. Coincidently, in both studies long shear displacements were imposed (> 2 m) and extensive decomposition were observed after the experiments.

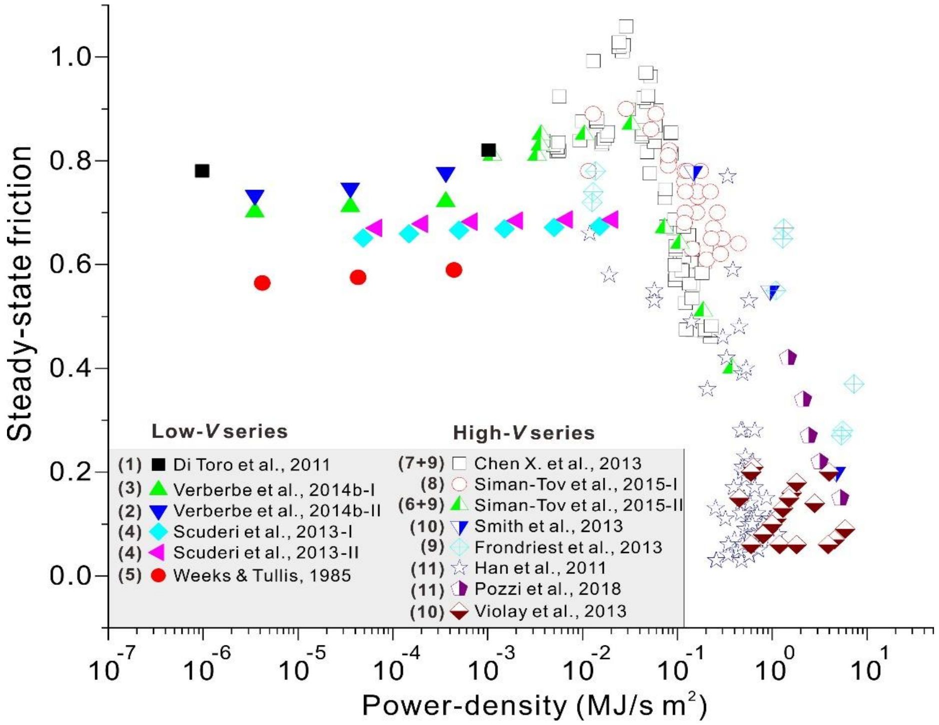

Figure S1. Compilation of steady-state friction coefficients of simulated fault gouges or bare surfaces of carbonate rocks, sheared at slip rates from 0.1 μm/s to 6.5 m/s under room humidity and temperature conditions. The data sets are the same as Figure 1 but now plotted as a function of power-density (PD = shear stress time slip velocity).

Text S2: Pre-exponential Constants in the Creep Equations

The least constrained parameters in the present model are the pre-exponential constants in the creep laws proposed, namely, A_n and A_t for the shear and compactional components, respectively (Eqs. 6a and 6b).

A). The Relation Between A_t and A_n

The first-order estimation between A_t and A_n can be made based on the geometry effect, assuming the deformation is by thermally-activated grain boundary (or solid-state) diffusion creep. With a representative microstructure for a granular fault gouge (Figure S1), we assume an arbitrary grain packing configuration under shearing with a porosity φ, corresponding to a dilatation angle ψ. For grain-scale creep by grain boundary diffusion, the rate-controlling process is the rate of mass removal at the grain contacts. In this configuration, the mass required to move in the normal direction is tanψ of that of the shear direction for the same amount of strain ($\Delta\gamma = \Delta\varepsilon$). As a first-order approximation, the geometry constants for creep in the two components are related as

$$A_n/A_t \approx tan\psi. \quad \text{(S1)}$$

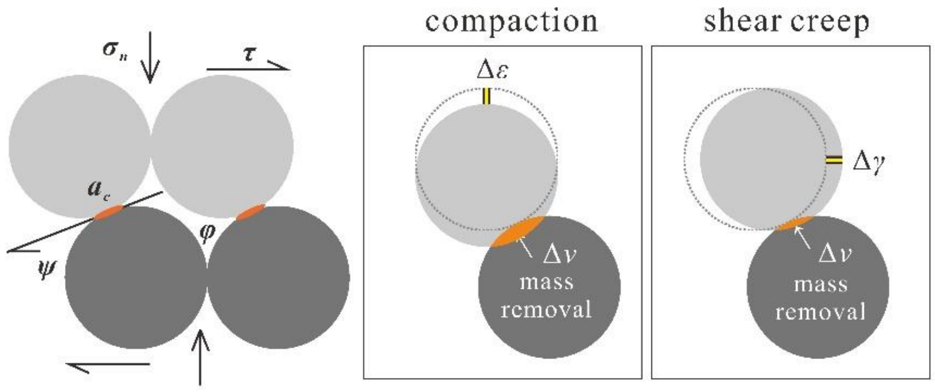

Figure S2. Diagram showing the difference in the mass move (Δv) for the same strain of deformation in the normal and shear directions ($\Delta\varepsilon = \Delta\gamma$), provided that the deformation is rate controlled by a grain boundary diffusion process.

As demonstrated by Ashby & Verral (1973), the mass move involved in GBS with full accommodation by diffusion is 1/7 of that required in classical diffusional flow, rendering a 7-times larger pre-exponential constant ($A_t = 7A$). As shown in Figure 10B, in the simple shear case, GBS with diffusion accommodation can be decomposed into three deformation steps: pure shear with dilatation, pure shear with compaction, and slight grain rotation. Assuming that grain rotation consumes negligible energy, the rate-controlling process will be the mass move involved in pure shear steps, similar to that in the *A&V model*. Recall that *the A&V model* was derived for material under 'pure shear' extension deformation. In the CNS model, when porosity is zero, tanψ reaches the maximum (tanψ = $2H\varphi_c$, from eq. (3)). Substituting the parameter values (H = 0.57 and φ_c = 20%, Table 2) for a calcite gouge gives

$$A_t = 4.4A_n. \tag{S2}$$

Given the uncertainties in the parameter values, this relation is quantitatively compatible with the *A&V model*.

Strictly speaking, Eq. (S2) only applies to diffusion creep (n = 1). For simplicity, we used the same relation for GBS with accommodation by dislocation creep. This is justified by the simulation results that GBS with other non-linear accommodation processes such as dislocation creep has a negligible contribution to the deformation (<< 1%).

B. The Absolute Values of A_n and A_t

The absolute values of the pre-exponential constants can be obtained by comparison with the classical creep laws, which are derived from compression tests at HPT conditions where the testing materials are at nearly-zero porosity,

$$\dot{\varepsilon} = A\frac{\sigma^n}{d^m}exp\left(-\frac{E_a}{RT}\right). \tag{S3}$$

Here, σ is the (differential) flow stress and $\dot{\varepsilon}$ is the (compactional) strain rate for the compression tests. In the CNS model, when porosity is zero, the creep equation in the shear direction reduces to (let φ = 0 in Eq. 6)

$$\dot{\gamma}_{pl} = A_t\frac{\tau^n}{d^m}exp\left(-\frac{E_a}{RT}\right). \tag{S4}$$

To use the lab-derived flow law of the same form (cf. Eqs. S2 and S3), we converted the shear stress (τ) and shear strain rate ($\dot{\gamma}$) determined from a friction experiment into the (equivalent) flow stress (σ) and strain rate ($\dot{\varepsilon}$) for the compression tests (using the Von Mises equivalent stress), yielding $\sigma = \sqrt{3}\tau$ and $\dot{\varepsilon} = \dot{\gamma}/\sqrt{3}$. Substituting these relations into the lab-derived flow law gives $A_t = A\sqrt{3}^{n+1}$. Applying eq. (S1) to Eq. (2a) yields $A_n = 2\sqrt{3}^{n+1}H\varphi_c A$. Using the H and φ_c values for a calcite gouge and a lab-derived law (n = 1.7, Schmid et al., 1977), results in $A_n = 1.01A$.

Text S3: Mimicking the Velocity Profile Used in the HVF Experiments

The velocity profiles used in our simulations of HVF experiments simply mimic the reported in the experiments. For the experiments by De Pao et al. (2015), the input *V*-profile consists of a short acceleration phase, a constant-V sliding period at 1 m/s, and a final deceleration phase, resulting in a total displacement of ~1.5 m. Since the acceleration rate was not a constant in the experiments, we used an error function to generate a *V*-profile similar to the one reported in the paper (cf. Figure 7 and Figure 1 of De Paola et al., 2015). As for the experiments by Smith et al. (2015), we simulated a *V*-pulse with a maximum velocity of 2.3 m/s using a constant acceleration rate (2.87 m/s^2 for acceleration and -2.87 m/s^2 for deceleration). This generates a *V*-profile similar to the reported in the paper (cf. Figures 8A and 8B).

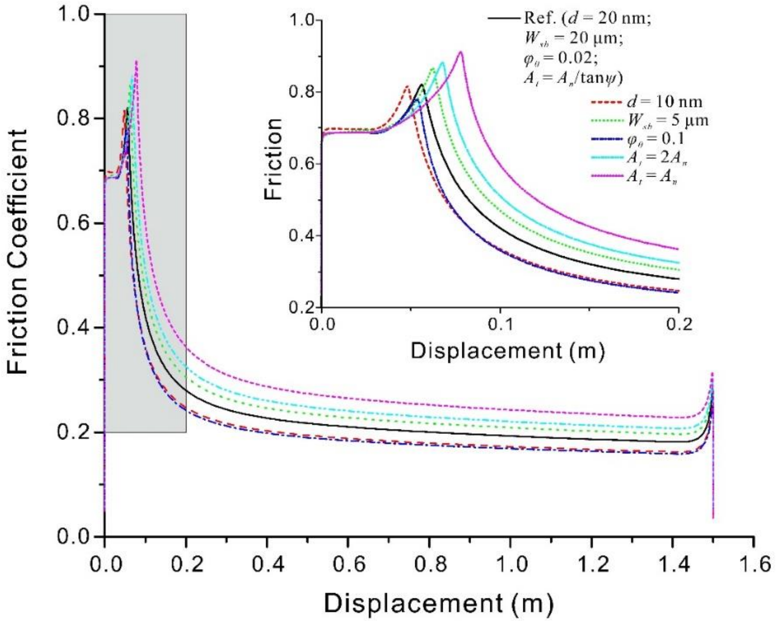

Figure S3. Sensitivity of the prediction friction curves to the parameter values. The reference case is to simulating De Paola et al.'s experiment at σ_n = 18 MPa. Here, we varied the PSZ grain size (*d*) and thickness (W_{sb}), the limited porosity (φ_0), and the pre-exponential constants in the creep laws (A_t/A_n). The inset gives a better illustration of the simulated results at short displacements.
